# Hyperoxemia and excess oxygen use in early acute respiratory distress syndrome: insights from the LUNG SAFE study

**DOI:** 10.1186/s13054-020-2826-6

**Published:** 2020-03-31

**Authors:** Fabiana Madotto, Emanuele Rezoagli, Tài Pham, Marcello Schmidt, Bairbre McNicholas, Alessandro Protti, Rakshit Panwar, Giacomo Bellani, Eddy Fan, Frank van Haren, Laurent Brochard, John G. Laffey, for the LUNG SAFE Investigators and the ESICM Trials Group, Antonio Pesenti, John G. Laffey, Laurent Brochard, Andres Esteban, Luciano Gattinoni, Frank van Haren, Anders Larsson, Daniel F. McAuley, Marco Ranieri, Gordon Rubenfeld, B. Taylor Thompson, Hermann Wrigge, Arthur S. Slutsky, Fernando Rios, T. Sottiaux, P. Depuydt, Fredy S. Lora, Luciano Cesar Azevedo, Eddy Fan, Guillermo Bugedo, Haibo Qiu, Marcos Gonzalez, Juan Silesky, Vladimir Cerny, Jonas Nielsen, Manuel Jibaja, Tài Pham, Dimitrios Matamis, Jorge Luis Ranero, Pravin Amin, S. M. Hashemian, Kevin Clarkson, Giacomo Bellani, Kiyoyasu Kurahashi, Asisclo Villagomez, Amine Ali Zeggwagh, Leo M. Heunks, Jon Henrik Laake, Jose Emmanuel Palo, Antero do Vale Fernandes, Dorel Sandesc, Yaasen Arabi, Vesna Bumbasierevic, Nicolas Nin, Jose A. Lorente, Lise Piquilloud, Fekri Abroug, Daniel F. McAuley, Lia McNamee, Javier Hurtado, Ed Bajwa, Gabriel Démpaire, Uhc Mother Theresa, Hektor Sula, Lordian Nunci, Alma Cani, Villa Maria, Alan Zazu, Christian Dellera, Carolina S. Insaurralde, Risso V. Alejandro, Julio Daldin, Mauricio Vinzio, Ruben O. Fernandez, Luis P. Cardonnet, Lisandro R. Bettini, Mariano Carboni Bisso, Emilio M. Osman, Mariano G. Setten, Pablo Lovazzano, Javier Alvarez, Veronica Villar, Norberto C. Pozo, Nicolas Grubissich, Gustavo A. Plotnikow, Daniela N. Vasquez, Santiago Ilutovich, Norberto Tiribelli, Ariel Chena, Carlos A. Pellegrini, María G. Saenz, Elisa Estenssoro, Matias Brizuela, Hernan Gianinetto, Pablo E. Gomez, Valeria I. Cerrato, Marco G. Bezzi, Silvina A. Borello, Flavia A. Loiacono, Adriana M. Fernandez, Serena Knowles, Claire Reynolds, Deborah M. Inskip, Jennene J. Miller, Jing Kong, Christina Whitehead, Shailesh Bihari, Aylin Seven, Amanda Krstevski, Helen J. Rodgers, Rebecca T. Millar, Toni E. Mckenna, Irene M. Bailey, Gabrielle C. Hanlon, Anders Aneman, Joan M. Lynch, Raman Azad, John Neal, Paul W. Woods, Brigit L. Roberts, Mark R. Kol, Helen S. Wong, Katharina C. Riss, Thomas Staudinger, Xavier Wittebole, Caroline Berghe, Pierre A. Bulpa, Alain M. Dive, Rik Verstraete, Herve Lebbinck, Pieter Depuydt, Joris Vermassen, Philippe Meersseman, Helga Ceunen, Jonas I. Rosa, Daniel O. Beraldo, Claudio Piras, Adenilton M. Rampinelli, Antonio P. Nassar, Sergio Mataloun, Marcelo Moock, Marlus M. Thompson, Claudio H. Gonçalves, Ana Carolina P. Antônio, Aline Ascoli, Rodrigo S. Biondi, Danielle C. Fontenele, Danielle Nobrega, Vanessa M. Sales, Suresh Shindhe, Dk Maizatul Aiman B. Pg Hj Ismail, John Laffey, Francois Beloncle, Kyle G. Davies, Rob Cirone, Venika Manoharan, Mehvish Ismail, Ewan C. Goligher, Mandeep Jassal, Erin Nishikawa, Areej Javeed, Gerard Curley, Nuttapol Rittayamai, Matteo Parotto, Niall D. Ferguson, Sangeeta Mehta, Jenny Knoll, Antoine Pronovost, Sergio Canestrini, Alejandro R. Bruhn, Patricio H. Garcia, Felipe A. Aliaga, Pamela A. Farías, Jacob S. Yumha, Claudia A. Ortiz, Javier E. Salas, Alejandro A. Saez, Luis D. Vega, Eduardo F. Labarca, Felipe T. Martinez, Nicolás G. Carreño, Pilar Lora, Haitao Liu, Ling Liu, Rui Tang, Xiaoming Luo, Youzhong An, Huiying Zhao, Yan Gao, Zhe Zhai, Zheng L. Ye, Wei Wang, Wenwen Li, Qingdong Li, Ruiqiang Zheng, Wenkui Yu, Juanhong Shen, Xinyu Li, Tao Yu, Weihua Lu, Ya Q. Wu, Xiao B. Huang, Zhenyang He, Yuanhua Lu, Hui Han, Fan Zhang, Renhua Sun, Hua X. Wang, Shu H. Qin, Bao H. Zhu, Jun Zhao, Jian Liu, Bin Li, Jing L. Liu, Fa C. Zhou, Qiong J. Li, Xing Y. Zhang, Zhou Li-Xin, Qiang Xin-Hua, Liangyan Jiang, Yuan N. Gao, Xian Y. Zhao, Yuan Y. Li, Xiao L. Li, Chunting Wang, Qingchun Yao, Rongguo Yu, Kai Chen, Huanzhang Shao, Bingyu Qin, Qing Q. Huang, Wei H. Zhu, Ai Y. Hang, Ma X. Hua, Yimin Li, Yonghao Xu, Yu D. Di, Long L. Ling, Tie H. Qin, Shou H. Wang, Junping Qin, Yi Han, Suming Zhou, Monica P. Vargas, Juan I. Silesky Jimenez, Manuel A. González Rojas, Jaime E. Solis-Quesada, Christian M. Ramirez-Alfaro, Jan Máca, Peter Sklienka, Jakob Gjedsted, Aage Christiansen, Boris G. Villamagua, Miguel Llano, Philippe Burtin, Gautier Buzancais, Pascal Beuret, Nicolas Pelletier, Satar Mortaza, Alain Mercat, Jonathan Chelly, Sébastien Jochmans, Nicolas Terzi, Cédric Daubin, Guillaume Carteaux, Nicolas de Prost, Jean-Daniel Chiche, Fabrice Daviaud, Tai Pham, Muriel Fartoukh, Guillaume Barberet, Jerome Biehler, Jean Dellamonica, Denis Doyen, Jean-Michel Arnal, Anais Briquet, Sami Hraiech, Laurent Papazian, Arnaud Follin, Damien Roux, Jonathan Messika, Evangelos Kalaitzis, Laurence Dangers, Alain Combes, Siu-Ming Au, Gaetan Béduneau, Dorothée Carpentier, Elie H. Zogheib, Herve Dupont, Sylvie Ricome, Francesco L. Santoli, Sebastien L. Besset, Philippe Michel, Bruno Gelée, Pierre-Eric Danin, Bernard Goubaux, Philippe J. Crova, Nga T. Phan, Frantz Berkelmans, Julio C. Badie, Romain Tapponnier, Josette Gally, Samy Khebbeb, Jean-Etienne Herbrecht, Francis Schneider, Pierre-Louis M. Declercq, Jean-Philippe Rigaud, Jacques Duranteau, Anatole Harrois, Russell Chabanne, Julien Marin, Charlene Bigot, Sandrine Thibault, Mohammed Ghazi, Messabi Boukhazna, Salem Ould Zein, Jack R. Richecoeur, Daniele M. Combaux, Fabien Grelon, Charlene Le Moal, Elise P. Sauvadet, Adrien Robine, Virginie Lemiale, Danielle Reuter, Martin Dres, Alexandre Demoule, Dany Goldgran-Toledano, Loredana Baboi, Claude Guérin, Ralph Lohner, Jens Kraßler, Susanne Schäfer, Kai D. Zacharowski, Patrick Meybohm, Andreas W. Reske, Philipp Simon, Hans-Bernd F. Hopf, Michael Schuetz, Thomas Baltus, Metaxia N. Papanikolaou, Theonymfi G. Papavasilopoulou, Giannis A. Zacharas, Vasilis Ourailogloy, Eleni K. Mouloudi, Eleni V. Massa, Eva O. Nagy, Electra E. Stamou, Ellada V. Kiourtzieva, Marina A. Oikonomou, Luis E. Avila, Cesar A. Cortez, Johanna E. Citalán, Sameer A. Jog, Safal D. Sable, Bhagyesh Shah, Mohan Gurjar, Arvind K. Baronia, Mohammedfaruk Memon, Radhakrishnan Muthuchellappan, Venkatapura J. Ramesh, Anitha Shenoy, Ramesh Unnikrishnan, Subhal B. Dixit, Rachana V. Rhayakar, Nagarajan Ramakrishnan, Vallish K. Bhardwaj, Heera L. Mahto, Sudha V. Sagar, Vijayanand Palaniswamy, Deeban Ganesan, Seyed Mohammadreza Hashemian, Hamidreza Jamaati, Farshad Heidari, Edel A. Meaney, Alistair Nichol, Karl M. Knapman, Donall O’Croinin, Eimhin S. Dunne, Dorothy M. Breen, Kevin P. Clarkson, Rola F. Jaafar, Rory Dwyer, Fahd Amir, Olaitan O. Ajetunmobi, Aogan C. O’Muircheartaigh, Colin S. Black, Nuala Treanor, Daniel V. Collins, Wahid Altaf, Gianluca Zani, Maurizio Fusari, Savino Spadaro, Carlo A. Volta, Romano Graziani, Barbara Brunettini, Salvatore Palmese, Paolo Formenti, Michele Umbrello, Andrea Lombardo, Elisabetta Pecci, Marco Botteri, Monica Savioli, Alessandro Protti, Alessia Mattei, Lorenzo Schiavoni, Andrea Tinnirello, Manuel Todeschini, Antonino Giarratano, Andrea Cortegiani, Sara Sher, Anna Rossi, Massimo M. Antonelli, Luca M. Montini, Paolo Casalena, Sergio Scafetti, Giovanna Panarello, Giovanna Occhipinti, Nicolò Patroniti, Matteo Pozzi, Roberto R. Biscione, Michela M. Poli, Ferdinando Raimondi, Daniela Albiero, Giulia Crapelli, Eduardo Beck, Vincenzo Pota, Vincenzo Schiavone, Alexandre Molin, Fabio Tarantino, Giacomo Monti, Elena Frati, Lucia Mirabella, Gilda Cinnella, Tommaso Fossali, Riccardo Colombo, Pierpaolo Terragni Ilaria Pattarino, Francesco Mojoli, Antonio Braschi, Erika E. Borotto, Andrea N. Cracchiolo, Daniela M. Palma, Francesco Raponi, Giuseppe Foti, Ettore R. Vascotto, Andrea Coppadoro, Luca Brazzi, Leda Floris, Giorgio A. Iotti, Aaron Venti, Osamu Yamaguchi, Shunsuke Takagi, Hiroki N. Maeyama, Eizo Watanabe, Yoshihiro Yamaji, Kazuyoshi Shimizu, Kyoko Shiozaki, Satoru Futami, Sekine Ryosuke, Koji Saito, Yoshinobu Kameyama, Keiko Ueno, Masayo Izawa, Nao Okuda, Hiroyuki Suzuki, Tomofumi Harasawa, Michitaka Nasu, Tadaaki Takada, Fumihito Ito, Shin - Nunomiya, Kansuke - Koyama, Toshikazu Abe, Kohkichi Andoh, Kohei Kusumoto, Akira Hirata, Akihiro Takaba, Hiroyasu Kimura, Shuhei Matsumoto, Ushio Higashijima, Hiroyuki Honda, Nobumasa Aoki, Hiroshi Imai, Yasuaki Ogino, Ichiko Mizuguchi, Kazuya Ichikado, Kenichi Nitta, Katsunori Mochizuki, Tomoaki Hashida, Hiroyuki Tanaka, Tomoyuki Nakamura, Daisuke Niimi, Takeshi Ueda, Yozo Kashiwa, Akinori Uchiyama, Olegs Sabelnikovs, Peteris Oss, Youssef Haddad, Kong Y. Liew, Silvio A. Ñamendys-Silva, Yves D. Jarquin-Badiola, Luis A. Sanchez-Hurtado, Saira S. Gomez-Flores, Maria C. Marin, Asisclo J. Villagomez, Jordana S. Lemus, Jonathan M. Fierro, Mavy Ramirez Cervantes, Francisco Javier Flores Mejia, Dulce Dector, Dulce M. Dector, Daniel R. Gonzalez, Claudia R. Estrella, Jorge R. Sanchez-Medina, Alvaro Ramirez-Gutierrez, Fernando G. George, Janet S. Aguirre, Juan A. Buensuseso, Manuel Poblano, Tarek Dendane, Hicham Balkhi, Mina Elkhayari, Nacer Samkaoui, Hanane Ezzouine, Abdellatif Benslama Mourad Amor, Wajdi Maazouzi, Nedim Cimic, Oliver Beck, Monique M. Bruns, Jeroen A. Schouten, Myra - Rinia, Monique Raaijmakers, Hellen M. Van Wezel, Serge J. Heines, Ulrich Strauch, Marc P. Buise, Fabienne D. Simonis, Marcus J. Schultz, Jennifer C. Goodson, Troy S. Browne, Leanlove Navarra, Anna Hunt, Robyn A. Hutchison, Mathew B. Bailey, Lynette Newby, Colin Mcarthur, Michael Kalkoff, Alex Mcleod, Jonathan Casement, Danielle J. Hacking, Finn H. Andersen, Merete S. Dolva, Jon H. Laake, Andreas Barratt-Due, Kim Andre L. Noremark, Eldar Søreide, Brit Å. Sjøbø, Anne B. Guttormsen, Hector H. Leon Yoshido, Ronald Zumaran Aguilar, Fredy A. Montes Oscanoa, Alain U. Alisasis, Joanne B. Robles, Rossini Abbie B. Pasanting-Lim, Beatriz C. Tan, Pawel Andruszkiewicz, Karina Jakubowska, Cristina M. Coxo, António M. Alvarez, Bruno S. Oliveira, Gustavo M. Montanha, Nelson C. Barros, Carlos S. Pereira, António M. Messias, Jorge M. Monteiro, Ana M. Araujo, Nuno T. Catorze, Susan M. Marum, Maria J. Bouw, Rui M. Gomes, Vania A. Brito, Silvia Castro, Joana M. Estilita, Filipa M. Barros, Isabel M. Serra, Aurelia M. Martinho, Dana R. Tomescu, Alexandra Marcu, Ovidiu H. Bedreag, Marius Papurica, Dan E. Corneci, Silvius Ioan Negoita, Evgeny Grigoriev, Alexey I. Gritsan, Andrey A. Gazenkampf, Ghaleb Almekhlafi, Mohamad M. Albarrak, Ghanem M. Mustafa, Khalid A. Maghrabi, Nawal Salahuddin, Tharwat M. Aisa, Ahmed S. Al Jabbary, Edgardo Tabhan, Yaseen M. Arabi, Olivia A. Trinidad, Hasan M. Al Dorzi, Edgardo E. Tabhan, Stefan Bolon, Oliver Smith, Jordi Mancebo, Hernan Aguirre-Bermeo, Juan C. Lopez-Delgado, Francisco Esteve, Gemma Rialp, Catalina Forteza, Candelaria De Haro, Antonio Artigas, Guillermo M. Albaiceta, Sara De Cima-Iglesias, Leticia Seoane-Quiroga, Alexandra Ceniceros-Barros, Antonio L. Ruiz-Aguilar, Luis M. Claraco-Vega, Juan Alfonso Soler, Maria del Carmen Lorente, Cecilia Hermosa, Federico Gordo, Miryam – Prieto-González, Juan B. López-Messa, Manuel P. Perez, Cesar P. Perez, Raquel Montoiro Allue, Ferran Roche-Campo, Marcos Ibañez-Santacruz, Susana - Temprano, Maria C. Pintado, Raul De Pablo, Pilar Ricart Aroa Gómez, Silvia Rodriguez Ruiz, Silvia Iglesias Moles, Mª. Teresa Jurado, Alfons Arizmendi, Enrique A. Piacentini, Nieves Franco, Teresa Honrubia, Meisy Perez Cheng, Elena Perez Losada, Javier - Blanco, Luis J. Yuste, Cecilia Carbayo-Gorriz, Francisca G. Cazorla-Barranquero, Javier G. Alonso, Rosa S. Alda, Ángela Algaba, Gonzalo Navarro, Enrique Cereijo, Esther Diaz-Rodriguez, Diego Pastor Marcos, Laura Alvarez Montero, Luis Herrera Para, Roberto Jimenez Sanchez, Miguel Angel Blasco Navalpotro, Ricardo Diaz Abad, Raquel Montiel Gonz á lez, D. á cil Parrilla Toribio, Alejandro G. Castro, Maria Jose D. Artiga, Oscar Penuelas, Tomas P. Roser, Moreno F. Olga, Elena Gallego Curto, Rocío Manzano Sánchez, Vallverdu P. Imma, Garcia M. Elisabet, Laura Claverias, Monica Magret, Ana M. Pellicer, Lucia L. Rodriguez, Jesús Sánchez-Ballesteros, Ángela González-Salamanca, Antonio G. Jimenez, Francisco P. Huerta, Juan Carlos J. Sotillo Diaz, Esther Bermejo Lopez, David D. Llinares Moya, Alec A. Tallet Alfonso, Palazon Sanchez Eugenio Luis, Palazon Sanchez Cesar, Sánchez I. Rafael, Corcoles G. Virgilio, Noelia N. Recio, Richard O. Adamsson, Christian C. Rylander, Bernhard Holzgraefe, Lars M. Broman, Joanna Wessbergh, Linnea Persson, Fredrik Schiöler, Hans Kedelv, Anna Oscarsson Tibblin, Henrik Appelberg, Lars Hedlund, Johan Helleberg, Karin E. Eriksson, Rita Glietsch, Niklas Larsson, Ingela Nygren, Silvia L. Nunes, Anna-Karin Morin, Thomas Kander, Anne Adolfsson, Hervé O. Zender, Corinne Leemann-Refondini, Souheil Elatrous, Slaheddine Bouchoucha, Imed Chouchene, Islem Ouanes, Asma Ben Souissi, Salma Kamoun, Oktay Demirkiran, Mustafa Aker, Emre Erbabacan, Ilkay Ceylan, Nermin Kelebek Girgin, Menekse Ozcelik, Necmettin Ünal, Basak Ceyda Meco, Onat O. Akyol, Suleyman S. Derman, Barry Kennedy, Ken Parhar, Latha Srinivasa, Danny McAuley, Phil Hopkins, Clare Mellis, Vivek Kakar, Dan Hadfield, Andre Vercueil, Kaushik Bhowmick, Sally K. Humphreys, Andrew Ferguson, Raymond Mckee, Ashok S. Raj, Danielle A. Fawkes, Philip Watt, Linda Twohey, Rajeev R. JhaMatthew Thomas, Alex Morton, Varsha Kadaba, Mark J. Smith, Anil P. Hormis, Santhana G. Kannan, Miriam Namih, Henrik Reschreiter, Julie Camsooksai, Alek Kumar, Szabolcs Rugonfalvi, Christopher Nutt, Orla O’Neill, Colette Seasman, Ged Dempsey, Christopher J. Scott, Helen E. Ellis, Stuart Mckechnie, Paula J. Hutton, Nora N. Di Tomasso, Michela N. Vitale, Ruth O. Griffin, Michael N. Dean, Julius H. Cranshaw, Emma L. Willett, Nicholas Ioannou, Sarah Gillis, Peter Csabi, Rosaleen Macfadyen, Heidi Dawson, Pieter D. Preez, Alexandra J. Williams, Owen Boyd, Laura Ortiz-Ruiz De Gordoa, Jon Bramall, Sophie Symmonds, Simon K. Chau, Tim Wenham, Tamas Szakmany, Piroska Toth-Tarsoly, Katie H. McCalman, Peter Alexander, Lorraine Stephenson, Thomas Collyer, Rhiannon Chapman, Raphael Cooper, Russell M. Allan, Malcolm Sim, David W. Wrathall, Donald A. Irvine, Kim S. Zantua, John C. Adams, Andrew J. Burtenshaw, Gareth P. Sellors, Ingeborg D. Welters, Karen E. Williams, Robert J. Hessell, Matthew G. Oldroyd, Ceri E. Battle, Suresh Pillai, Istvan Kajtor, Mageswaran Sivashanmugavel, Sinead C. O’Kane, Adrian Donnelly, Aniko D. Frigyik, Jon P. Careless, Martin M. May, Richard Stewart, T. John Trinder, Samantha J. Hagan, Matt P. Wise, Jade M. Cole, Caroline C. MacFie, Anna T. Dowling, Nicolás Nin, Edgardo Nuñez, Gustavo Pittini, Ruben Rodriguez, María C. Imperio, Cristina Santos, Ana G. França, Alejandro EBEID, Alberto Deicas, Carolina Serra, Aditya Uppalapati, Ghassan Kamel, Valerie M. Banner-Goodspeed, Jeremy R. Beitler, Satyanarayana Reddy Mukkera, Shreedhar Kulkarni, Jarone Lee, Tomaz Mesar, John O. Shinn, Dina Gomaa, Christopher Tainter, Dale J. Yeatts, Jessica Warren, Michael J. Lanspa, Russel R. Miller, Colin K. Grissom, Samuel M. Brown, Philippe R. Bauer, Ryan J. Gosselin, Barrett T. Kitch, Jason E. Cohen, Scott H. Beegle, Renaud M. Gueret, Aiman Tulaimat, Shazia Choudry, William Stigler, Hitesh Batra, Nidhi G. Huff, Keith D. Lamb, Trevor W. Oetting, Nicholas M. Mohr, Claine Judy, Shigeki Saito, Fayez M. Kheir, Fayez Kheir, Adam B. Schlichting, Angela Delsing, Daniel R. Crouch, Mary Elmasri, Dina Ismail, Kyle R. Dreyer, Thomas C. Blakeman, Rebecca M. Baron, Carolina Quintana Grijalba, Peter C. Hou, Raghu Seethala, Imo Aisiku, Galen Henderson, Gyorgy Frendl, Sen-Kuang Hou, Robert L. Owens, Ashley Schomer, Vesna Bumbasirevic, Bojan Jovanovic, Maja Surbatovic, Milic Veljovic

**Affiliations:** 1 Research Center on Public Health, School of Medicine and Surgery, University of Milano-Bicocca, Monza, Italy; 2 Scientific Institute for Research, Hospitalization and Health Care, IRCCS Multimedica, Sesto San Giovanni, Milan, Italy; 3 Department of Medicine and Surgery, University of Milano-Bicocca, Monza, Italy; 4 Anaesthesia and Intensive Care Medicine, School of Medicine, National University of Ireland Galway, Galway, Ireland; 5 Regenerative Medicine Institute (REMEDI) at CÚRAM Centre for Research in Medical Devices, Biomedical Sciences Building, National University of Ireland Galway, Galway, Ireland; 6 Keenan Research Centre for Biomedical Science, St Michael’s Hospital, Toronto, Canada; 7 Department of Critical Care Medicine, St Michael’s Hospital, Toronto, Canada; 8 Interdepartmental Division of Critical Care Medicine, University of Toronto, Toronto, Canada; 9 Institute of Health Policy, Management and Evaluation, University of Toronto, Toronto, Canada; 10 Nephrology, School of Medicine, National University of Ireland Galway, Galway, Ireland; 11 Department of Biomedical Sciences, Humanitas University, Pieve Emanuele (Milan), Italy; 12 Humanits clinical and research center – IRCCS, Rozzano (Milan), Italy; 13 Intensive Care Unit, John Hunter Hospital, New Lambton Heights, NSW Australia; 14 School of Medicine and Public Health, University of Newcastle, Newcastle, Australia; 15 Department of Emergency and Intensive Care, San Gerardo Hospital, Monza, Italy; 16 Department of Medicine, University Health Network and Sinai Health System, Toronto, Canada; 17 Intensive Care Unit, The Canberra Hospital and Australian National University, Canberra, Australia

**Keywords:** Hyperoxia, Hypoxia, Hyperoxemia, Hypoxemia, Oxygen therapy, Acute respiratory distress syndrome, Mortality, Invasive mechanical ventilation

## Abstract

**Background:**

Concerns exist regarding the prevalence and impact of unnecessary oxygen use in patients with acute respiratory distress syndrome (ARDS). We examined this issue in patients with ARDS enrolled in the *L*arge observational study to *UN*derstand the *Gl*obal impact of *S*evere *A*cute respiratory *F*ailur*E* (LUNG SAFE) study.

**Methods:**

In this secondary analysis of the LUNG SAFE study, we wished to determine the prevalence and the outcomes associated with hyperoxemia on day 1, sustained hyperoxemia, and excessive oxygen use in patients with early ARDS. Patients who fulfilled criteria of ARDS on day 1 and day 2 of acute hypoxemic respiratory failure were categorized based on the presence of hyperoxemia (PaO_2_ > 100 mmHg) on day 1, sustained (i.e., present on day 1 and day 2) hyperoxemia, or excessive oxygen use (FIO_2_ ≥ 0.60 during hyperoxemia).

**Results:**

Of 2005 patients that met the inclusion criteria, 131 (6.5%) were hypoxemic (PaO_2_ < 55 mmHg), 607 (30%) had hyperoxemia on day 1, and 250 (12%) had sustained hyperoxemia. Excess FIO_2_ use occurred in 400 (66%) out of 607 patients with hyperoxemia. Excess FIO_2_ use decreased from day 1 to day 2 of ARDS, with most hyperoxemic patients on day 2 receiving relatively low FIO_2_. Multivariate analyses found no independent relationship between day 1 hyperoxemia, sustained hyperoxemia, or excess FIO_2_ use and adverse clinical outcomes. Mortality was 42% in patients with excess FIO_2_ use, compared to 39% in a propensity-matched sample of normoxemic (PaO_2_ 55–100 mmHg) patients (*P* = 0.47).

**Conclusions:**

Hyperoxemia and excess oxygen use are both prevalent in early ARDS but are most often non-sustained. No relationship was found between hyperoxemia or excessive oxygen use and patient outcome in this cohort.

**Trial registration:**

LUNG-SAFE is registered with ClinicalTrials.gov, NCT02010073

## Key messages

- Hyperoxemia and excess FIO_2_ use was prevalent in patients with early ARDS. Hyperoxemia occurred in 30% of patients, while two thirds of these patients received excess oxygen therapy.
- While a similar proportion of patients were hyperoxemic on day 2 of ARDS, higher FIO_2_ use did decrease. Consequently, most day 2 hyperoxemia was seen in patients at lower FIO_2_, in whom gas exchange was improving.
- In the majority of patients, both hyperoxemia and excess oxygen use were transient, although sustained hyperoxemia occurred in 12% of patients.
- Higher FIO_2_ use was independently associated with the risk of hyperoxemia, illustrating the need for close attention to oxygen use to reduce this risk.
- We found no relationship between the degree and duration of hyperoxemia or of excessive oxygen use, and outcome in early ARDS, in this patient cohort.

## Background

Acute respiratory distress syndrome (ARDS) is a syndrome characterized by impaired gas exchange resulting in low oxygen tensions in the blood (i.e., hypoxemia) and tissues (i.e., hypoxia) [1]. Tissue hypoxia is harmful, leading to cell death, organ failure, and increased mortality in the critically ill [2]. While oxygen therapy can reverse tissue hypoxia, little evidence exists regarding the optimal use of oxygen in patients with ARDS. Critically ill patients frequently receive higher inspired oxygen concentrations than necessary [3], perhaps due to concerns regarding tissue hypoxia [4, 5].

Hyperoxemia and the resultant tissue hyperoxia may worsen systemic organ injury in the critically ill. Arterial hyperoxemia has been associated with increased mortality in some older [6–8] but not more recent [9, 10] studies of patients with acute brain injury. Hyperoxemia was associated with worse outcomes in cohort patients with acute ischemic stroke or subarachnoid/intracerebral hemorrhage that required invasive mechanical ventilation [11]. Supplemental oxygen therapy worsened myocardial injury and infarct size in patients post myocardial infarction [12]. In patients resuscitated post cardiac arrest, hyperoxia has been associated with harm in several [13–16] studies, although the most recent study [17] did not confirm this. Potential mechanisms of oxygen toxicity remain poorly understood and may include systemic arterial vasoconstriction [18, 19], and cytotoxic effects of reactive oxygen species [20–22]. In randomized trials, “induced” hyperoxia (using 100% oxygen) increased 28-day mortality in septic shock patients [23], while critically ill patients randomized to a target arterial oxygen tension (PaO_2_) of 70–100 mmHg had lower mortality compared to patients with a “conventional” target of PaO_2_ up to 150 mmHg [24] in a single-center study. While a recent large international multicenter trial demonstrated no effect of conservative oxygen therapy in a diverse cohort of critically ill patients [25], a subsequent sub-study raised the possibility of clinically important harm with conservative oxygen therapy in patients with sepsis [26].

In ARDS, the relationship between oxygen use and outcome is complex. The severely impaired gas exchange means that high fraction of inspired oxygen (FIO_2_) use may simply reflect a more severe alveolar-arterial oxygen gradient and hence be a marker of ARDS severity. In mild ARDS, relatively modest levels of FIO_2_ may result in (moderate) hyperoxemia and tissue hyperoxia. In addition, severe degrees of systemic hyperoxemia (i.e., PaO_2_ > 300) associated with harm in other critically ill populations are not possible in ARDS. However, even moderate systemic hyperoxemia that may be more commonly seen in ARDS could be harmful [27]. Furthermore, the use of high FIO_2_ can have direct toxic effects on the lung [28, 29], sensitize the lung to subsequent injury, adversely affect the lung innate immune response [30], and worsen ventilation-induced injury [31–33]. These complexities highlight the need to distinguish between hyperoxemia and high FIO_2_ use. In patients receiving high FIO_2_, it is important to determine whether this was necessary to achieve normoxemia or if it could have been avoided (i.e., excess oxygen use).

We wished to examine the impact of hyperoxemia and of excess oxygen use in this secondary analysis of patients with ARDS in the LUNG SAFE patient cohort [34]. Our primary objective was to determine the prevalence of early and sustained hyperoxemia and of excess oxygen use in patients with hyperoxemia. Secondary objectives included identifying factors associated with hyperoxemia and with excess oxygen use and examining the relationship between hyperoxemia and excess oxygen use and outcomes from ARDS.

## Methods

### Design, setting, and participants

This is a sub-study of the LUNG SAFE study, an international, multicenter, prospective cohort study of patients receiving invasive or noninvasive ventilation, and the detailed methods and protocol have been published elsewhere [34]. In brief, LUNG SAFE was an international, multicenter, prospective cohort study, with a 4-week enrolment window in the winter season in both hemispheres [34]. National coordinators and site investigators obtained ethics committee approval and ensured data integrity and validity.

**Fig. 1:**
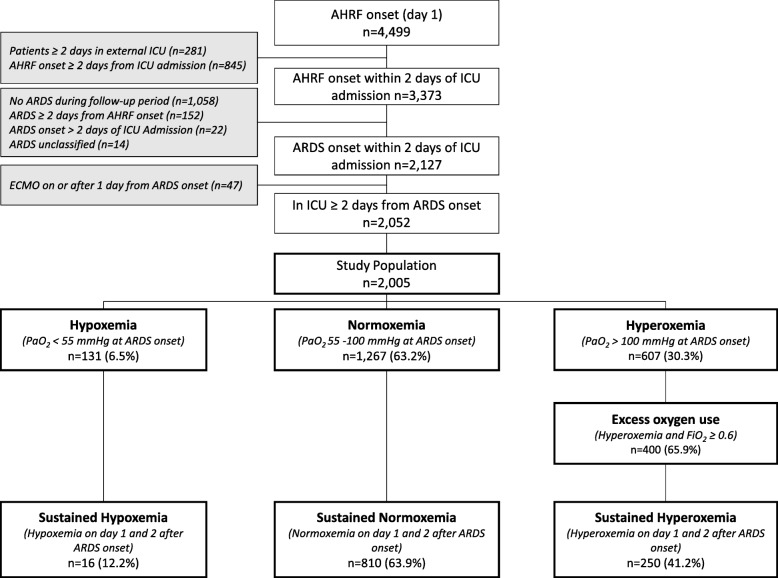
Flow chart describing criteria used to select and to classify the ARDS study population

Given the study focus on early hyperoxemia and excess oxygen use, we restricted the study population to patients that fulfilled ARDS criteria within 48 h of ICU admission, and who remained in the ICU for at least 2 days from ARDS onset. Patients transferred from other ICUs after 2 days, patients that developed ARDS later in their ICU stay, and patients that received early ECMO were excluded (Fig. 1). Additional methodological details are available in Additional file 1.

### Data collection and analysis

All data were recorded for each patient at the same time each day within participating ICUs, normally as close as possible to 10 a.m. each day. Data on ventilatory settings were recorded simultaneously with arterial blood gas analysis. The following definitions were applied on day 1 and on day 2 of ARDS: hypoxemia (PaO_2_ < 55 mmHg), normoxemia (PaO_2_ 55–100 mmHg), and hyperoxemia (PaO_2_ > 100 mmHg). Excess oxygen use was defined as the use of FIO_2_ ≥ 0.6 in patients with hyperoxemia (PaO_2_ > 100 mmHg). Patients with hyperoxemia on days 1 and 2 of ARDS were considered to have sustained hyperoxemia. Analogously, we also defined patients with sustained hypoxemia and sustained normoxemia.

The duration of invasive mechanical ventilation (MV) was calculated as the number of days between the date of intubation and the date of extubation in ICU (or death, if the patient died under invasive MV). Similarly, invasive ventilator-free days were calculated as the number of days from weaning from invasive MV to day 28, and for patients who died before weaning, we considered to have a ventilator-free-day value of 0. Patient survival was evaluated at hospital discharge, or at day 90, whichever occurred first. Our other data definitions have been previously reported [34–37].

### Statistical analyses

Descriptive statistics included proportions for categorical and mean (standard deviation) or median (interquartile range) for continuous variables. No assumptions were made for missing data. To assess differences among three groups (systemic hypoxemia, normoxemia, and hyperoxemia), we performed chi-squared test (or Fisher exact test) for discrete variables and analysis of variance (ANOVA) (or Kruskal-Wallis test) for continuous variables. Bonferroni correction was applied to determine significance in the setting of multiple comparisons. Chi-square test (or Fisher exact test), Student’s *t* test (or Wilcoxon Mann Whitney test) were used to assess differences between groups (i.e., sustained hyperoxemia and sustained normoxemia) in discrete and continuous distributions of parameters, respectively.

Locally estimated scatterplot smoothing (LOESS) method was used to inspect the relationship between mortality and PaO_2_ and FIO_2_ measured on day 1 and on day 2 of ARDS.

Multivariable logistic regression models were used to evaluate factors associated with the presence of either hyperoxemia or excess of oxygen use, and with mortality. In each regression model, the independent predictors (demographic characteristics and clinical parameters measured at the first day of ARDS) were identified through a stepwise regression approach. This approach combines forward and backward selection methods in an iterative procedure (with a significance level of 0.05 both for entry and retention) to select predictors in the final multivariable model. Results were reported as odds ratio (OR) with 95% confidence interval (CI).

Propensity score matching method was applied to evaluate the possible impact of sustained hyperoxemia on main outcomes (mortality, ventilation-free days, and duration of MV) in patients with mild-moderate ARDS. Patients with severe ARDS were excluded as there were no such patients in the sustained hyperoxemia group. In detail, patients with sustained hyperoxemia and sustained normoxemia were matched (1:1 match without replacement), using a caliper of 0.2 standard deviation of the logit of the propensity score, and the balance between the matched groups was assessed by the standardized differences of each independent variable used in the propensity score estimation. Statistical significance of the difference in continuous variables, as ventilation-free days and duration of MV, was evaluated with Wilcoxon signed-rank test, while for difference in proportions of deaths, we applied McNemar’s test. Survival probability in these matched groups was estimated using the Kaplan-Meier approach and assuming that patients discharged alive from hospital before 90 days were alive on day 90. Statistical difference between survival curves was assessed through Kein and Moeschberger test. The same approach was used to assess the possible impact of excess use of oxygen on main outcomes.

All *p* values were two-sided, with *p* values < 0.05 considered as statistically significant. Statistical analyses were performed with R, version 3.5.2. (R Project for Statistical Computing, http://www.R-project.org) and SAS software, version 9.4 (SAS Institute, Cary, NC, USA).

## Results

Of 4499 patients that developed AHRF in the LUNG SAFE cohort, 2127 of these developed ARDS within 2 days of ICU admission, of whom 2052 remained in ICU for at least 2 days from ARDS onset. The study population consists of 2005 of these patients that did not receive ECMO (Fig. 1).

### Systemic oxygen tensions

**Fig. 2:**
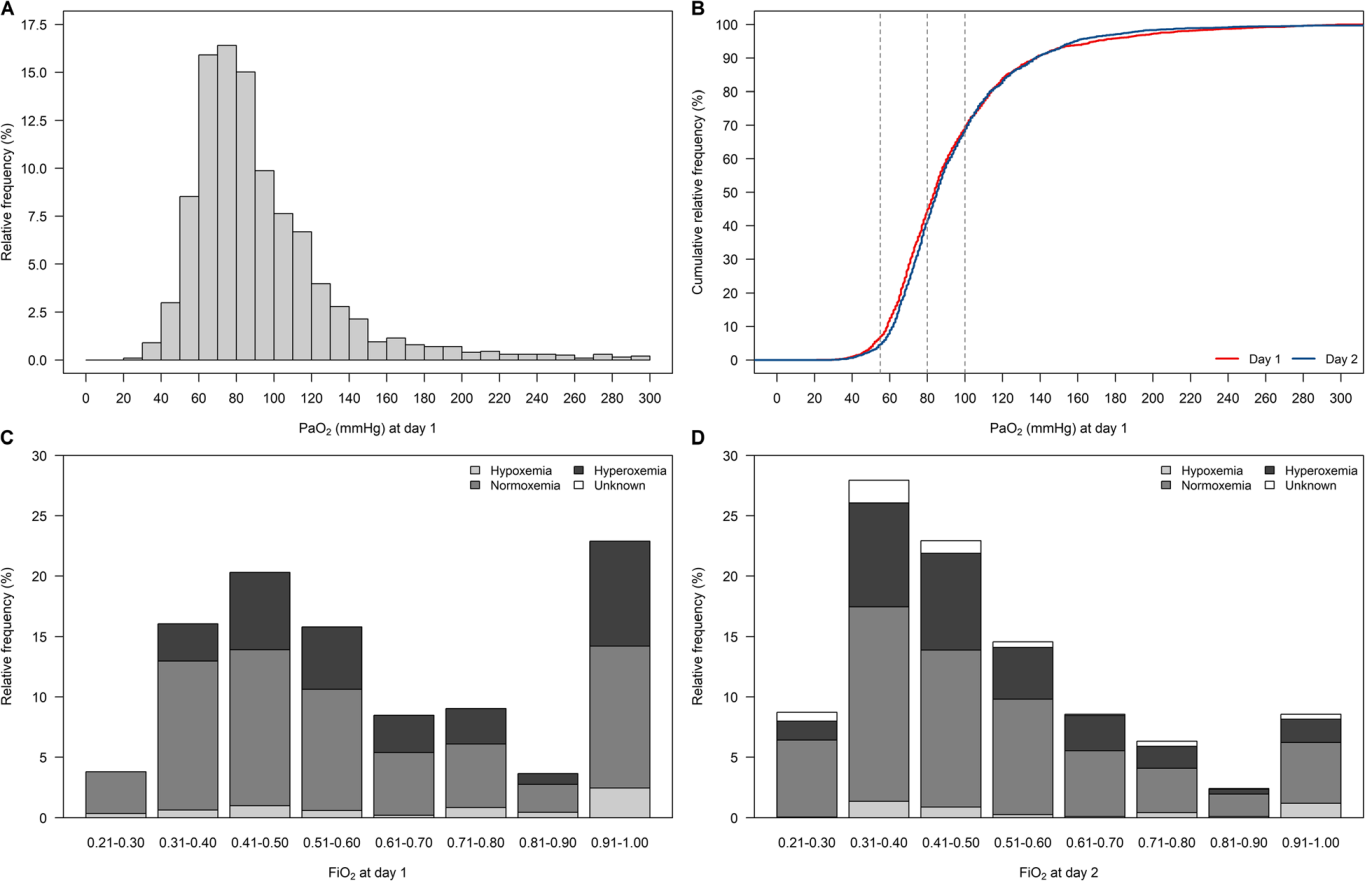
Arterial oxygen tensions and use of oxygen in patients on days 1 and 2 of ARDS. **a** The distribution of PaO_2_ on day 1 of ARDS, demonstrating a wide range of PaO_2_. **b** Density distributions of PaO_2_ on days 1 (red line) and day 2 (blue line) of ARDS. **c** Histogram of FIO_2_ and PaO_2_ on day 1 of ARDS. **d** Histogram of FIO_2_ and PaO_2_ on day 2 of ARDS. Note: in **c** and **d**, each bar is segmented into hyperoxemia (black), normoxemia (dark gray), hypoxemia (light gray), and unknown (white) component

In the study population, 607 subjects (30%) were hyperoxemic, while 6.5% of patients remained hypoxemic, on day 1 of ARDS (Fig. 2a, Table 1, eTable 1). Density distributions of arterial oxygen tension on days 1 and 2 of ARDS (Fig. 2b) reveal similar PaO_2_ profiles for days 1 and 2. In the hyperoxemic population at day 1, 59% had a transient hyperoxemia, while in 250 (41%) patients, the condition was sustained, with PaO_2_ > 100 mmHg on both the first and second day of ARDS (Fig. 1; eTable 2). All eTables are included in Additional file 1.

**Table 1:** Characteristics of study population (*n* = 2005), stratified by arterial oxygenation on day 1

Parameter	Hypoxemia (PaO_2_ < 55 mmHg)	Normoxemia (55 mmHg ≤ PaO_2_ ≤ 100 mmHg)	Hyperoxemia (PaO_2_ > 100 mmHg)	*p* value (among groups)
*N* (%)	131 (6.53)	1267 (63.19)	607 (30.27)	
Male, *n* (%)	73 (55.73)	796 (62.83)	359 (59.14)	0.1259
Age (years), mean ± SD	59.20 ± 16.86	62.21 ± 16.74	61.88 ± 16.82	0.1264
BMI (kg/m^2^), mean ± SD	27.23 ± 6.82	27.66 ± 8.26	26.90 ± 6.84	0.5646
**ARDS risk factors**, ***n*** (**%**)				
None	9 (6.87)	103 (8.13)	52 (8.57)	0.8088
Only non-pulmonary	15 (11.45)	229 (18.07)	106 (17.46)	0.1641
Only pulmonary	92 (70.23)	769 (60.62)	357 (58.81)	0.0525
Both	15 (11.45)	167 (13.18)	92 (15.16)	0.3789
**Illness severity at ARDS onset**				
P_a_O_2_ (mmHg), mean ± SD	47.1 ± 6.2	76.8 ± 11.9*	137.8 ± 41.0*^†^	< 0.0001
P_a_O_2_/FIO_2_ (mmHg), mean ± SD	75.12 ± 38.97	140.49 ± 56.70*	205.90 ± 54.88*^†^	< 0.0001
SpO_2_ (%), median (q_1_–q_3_)	88 (82–94)	95 (92–97)*	98 (97–99)*^†^	< 0.0001
ARDS severity, *n* (%)				< 0.0001
Mild	4 (3.05)	205 (16.18)*	330 (54.37)*^†^	< 0.0001
Moderate	22 (16.79)	683 (53.91)*	273 (33.98)*^†^	< 0.0001
Severe	105 (80.15)	379 (29.91)*	4 (0.66)*^†^	< 0.0001
P_a_CO_2_ (mmHg), mean ± SD	54.3 ± 25.0	46.3 ± 15.3*	44.8 ± 14.6*	0.0031
pH, mean ± SD	7.31 ± 0.15	7.33 ± 0.12	7.32 ± 0.13	0.7446
Bicarbonate (mmol/L), mean ± SD	26.3 ± 10.9	23.3 ± 6.5	22.3 ± 6.3*^†^	< 0.0001
Base excess (mEq/L), mean ± SD	0.5 ± 10.8	−2.0 ± 6.8	−3.1 ± 6.8*^†^	0.0001
Non-respiratory SOFA score adjusted, mean ± SD	6.14 ± 4.15	6.08 ± 3.94	6.28 ± 4.00	0.6246
SOFA score adjusted, mean ± SD	10.25 ± 4.20	9.51 ± 3.98	8.87 ± 3.93*^†^	0.0005
Respiration	3.79 ± 0.46	3.15 ± 0.66	2.45 ± 0.50	< 0.0001
Central nervous system	1.68 ± 1.72	1.74 ± 1.66	1.92 ± 1.69	0.1161
Cardiovascular	1.77 ± 1.74	2.03 ± 1.76	1.93 ± 1.74	0.1760
Liver	0.65 ± 0.98	0.54 ± 0.96	0.51 ± 0.92	0.2855
Coagulation	1.20 ± 1.45	0.95 ± 1.31	0.98 ± 1.34	0.1751
Renal	0.69 ± 1.04	1.77 ± 1.10	0.88 ± 1.22	0.3333
Pressor support infusion rates				
Dopamine (μg/kg/min), mean ± SD	8.19 ± 6.94	8.58 ± 5.02	7.73 ± 5.98	0.4151
Dobutamine (μg/kg/min), mean ± SD	5.52 ± 3.74	5.72 ± 4.05	6.75 ± 3.53	0.3565
Noradrenaline (μg/kg/min), mean ± SD	0.50 ± 0.67	0.45 ± 0.75	0.54 ± 1.48	0.6701
Adrenaline (μg/kg/min), mean ± SD	1.10 ± 2.03	0.48 ± 0.69	0.43 ± 0.55	0.8434
**Management factors at ARDS onset**				
Invasive mechanical ventilation, *n* (%)	102 (77.86)	1000 (78.93)	506 (83.36)	0.0619
Control mode of ventilation, mean ± SD	70 (54.69)	693 (55.89)	386 (64.23)^†^	0.0021
FIO_2_, median (q_1_–q_3_)	0.80 (0.50–1.00)	0.60 (0.41–0.80)*	0.65 (0.50–1.00)^†^	< 0.0001
FIO_2_ ≥ 0.6, *n* (%)	90 (68.70)	670 (52.88)*	400 (65.90)^†^	< 0.0001
FIO_2_ ≥ 0.6 at 2nd day, *n* (%)^‡^	64 (73.56)	372 (57.94)*	167 (43.60)*^†^	< 0.0001
Tidal volume (ml/kg), mean ± SD	7.9 ± 2.2	7.8 ± 2.0	7.9 ± 2.0	0.2256
PEEP (cmH_2_O), mean ± SD	8.7 ± 3.33	8.1 ± 3.2	7.9 ± 3.1*	0.0174
PIP (cmH_2_O), mean ± SD	25.7 ± 8.90	25.3 ± 8.5	25.6 ± 8.7	0.6295
Dynamic compliance (ml/cmH_2_O), mean ± SD	39.0 ± 38.4	36.9 ± 37.8	35.6 ± 38.9	0.7759
Total respiratory rate (breaths/min), mean ± SD	23.2 ± 7.1	21.9 ± 6.9	21.1 ± 7.0	0.0003
Standardized minute ventilation (L/min), mean ± SD	14.4 ± 7.8	11.4 ± 5.3*	10.8 ± 5.0*^†^	< 0.0001
Patients in whom plateau pressure measured, *n* (%)°	24 (18.32)	304 (23.99)	186 (30.64)	0.0012
Plateau pressure (cmH_2_O), mean ± SD	24.3 ± 9.0	23.4 ± 6.1	23.0 ± 5.6	0.7512
Driving pressure (cmH_2_O), mean ± SD	16.0 ± 8.2	14.6 ± 5.4	15.0 ± 5.2	0.4941
**Clinical outcomes**				
Hospital mortality (90 days), *n* (%)	47 (35.88)	486 (38.54)	227 (37.52)	0.7934
Ventilation free days (days), median (q_1_–q_3_)				
All	10.0 (0.0–22.0)	12.0 (0.0–23.0)	16.0 (0.0–24.0)^†^	0.0303
Survivors at ICU discharge	20.0 (14.0–24.0)	21.0 (15.0–25.0)	23.0 (18.0–26.0)*^†^	0.0002
Duration mechanical ventilation (days), median (q_1_–q_3_)				
All	7.0 (4.0–13.0)	8.0 (4.0–15.0)	7.0 (3.0–13.0)^†^	0.0074
Survivors at ICU discharge	9.0 (5.0–15.0)	8.0 (4.0–14.0)	6.0 (3.0–11.0)*^†^	0.0002

*Abbreviations*: *ARDS* acute respiratory distress syndrome, *BMI* body mass index, *COPD* chronic obstructive pulmonary disease, *FIO*_*2*_ fraction of inspired oxygen, *P*_*a*_*O*_*2*_ arterial oxygen partial pressure, *P*_*a*_*CO*_*2*_ arterial carbon dioxide partial pressure, *PEEP* positive end-expiratory pressure, *PIP* peak inspiratory pressure, *q*_*1*_ first quartile, *q*_*3*_ third quartile, *SOFA* sepsis-related organ failure assessment, *SD* standard deviation, *SpO*_*2*_ peripheral oxygen saturation

°Plateau pressure and driving pressure values are limited to patients in whom this value was reported and in whom either an assist control mode was used or in whom a mode permitting spontaneous ventilation was used and where the set and total respiratory rates were equal. Patients receiving HFOV or ECMO were also excluded

^‡^Percentage was calculated on patients with FIO_2_ available during the second day and with FIO_2_ ≥ 0.60 at day 1

*p value < 0.05 (Bonferroni’s correction), comparison with “Hypoxemia” group

^†^*p* value < 0.05 (Bonferroni’s correction), comparison with “Normoxemia” group

A multivariable analysis of factors independently associated with day 1 hyperoxemia identified higher FIO_2_ use, lower PEEP, lower respiratory rate, a lower sepsis-related organ failure assessment (SOFA) cardiovascular score, and comorbidities such as neoplasm and/or immunosuppression and heart failure (Table 2).

**Table 2:** Factors associated with day 1 hyperoxemia (PaO_2_ > 100 mmHg) and with excess oxygen use (FIO_2_ ≥ 0.6 in patients with P_a_O_2_ > 100 mmHg) in the study population

Parameter	Odds ratio (95% confidence interval)	*p* value
**Outcome—hyperoxemia at day 1** (model* on 1855 patients)		
FIO_2_ (0.1 unit)	1.168 (1.115; 1.224)	< .0001
Bicarbonate (mmol/L)	0.967 (0.951; 0.984)	< .0001
Total respiratory rate (breath/min)	0.971 (0.956; 0.986)	0.0002
PEEP (cmH_2_O)	0.944 (0.910; 0.979)	0.0017
Active/hematologic neoplasm or immunosuppression (ref. no.)	1.414 (1.111; 1.801)	0.0050
SOFA score – Cardiovascular	0.925 (0.870; 0.984)	0.0139
Heart failure (ref. no.)	1.482 (1.080; 2.033)	0.0148
**Outcome—excess oxygen use at day 1** (**model**^**°**^**on 1694 patients**)		
P_a_O_2_/FIO_2_ (mmHg)	0.978 (0.976; 0.980)	< .0001
PEEP (cmH_2_O)	1.144 (1.091; 1.199)	< .0001
PIP (cmH_2_O)	1.029 (1.014; 1.045)	0.0002
Bicarbonate (mmol/L)	0.971 (0.954; 0.989)	0.0013
Age (years)	0.988 (0.981; 0.996)	0.0023
BMI (kg/m^2^)	0.980 (0.965; 0.995)	0.0111
Tidal volume (ml/kg IBW)	1.081 (1.017; 1.149)	0.0122

*Abbreviations*: *BMI* body mass index, *FIO*_*2*_ fraction of inspired oxygen, *PEEP* positive end-expiratory pressure, *PIP* peak inspiratory pressure, *SOFA* sepsis-related organ failure, *P*_*a*_*O*_*2*_ arterial oxygen partial pressure, *IBW* ideal body weight

*Multivariable logistic model with presence of hyperoxemia (PaO_2_ > 100 mmHg) as dependent dichotomous variable and the predictors were identified by stepwise approach. One hundred and fifty patients were excluded due to missing values for the response or explanatory variables. List of possible predictors in stepwise approach: age, sex, body mass index, comorbidities (presence of heart failure, diabetes mellitus chronic renal failure, chronic obstructive pulmonary disease or home ventilation, active neoplasm of hematologic neoplasm or immunosuppression), ARDS risk factors (none, only non-pulmonary, only pulmonary, both types), bicarbonates concentration, management factors (presence of invasive mechanical ventilation, tidal volume, PEEP, PIP, total respiratory rate, minute ventilation), and FIO_2_ and SOFA components (CNS, cardiovascular, renal, liver, coagulation score)

°Multivariable logistic model with excess of oxygen use (FIO_2_ ≥ 0.6 and PaO_2_ > 100 mmHg) as dependent dichotomous variable and predictors identified by stepwise approach. Three hundred and eleven observations were deleted due to missing values for the response or explanatory variables

List of possible predictors in stepwise approach: age, sex, body mass index, comorbidities (presence of heart failure, diabetes mellitus chronic renal failure, chronic obstructive pulmonary disease or home ventilation, active neoplasm of hematologic neoplasm or immunosuppression), ARDS risk factors (none, only non-pulmonary, only pulmonary, both types), bicarbonates concentration, management factors (presence of invasive mechanical ventilation, tidal volume, PEEP, PIP, total respiratory rate, minute ventilation), and PaO_2_/FIO_2_ ratio and non-respiratory SOFA components (CNS, cardiovascular, renal, liver, coagulation score)

### Use of oxygen

**Fig. 3:**
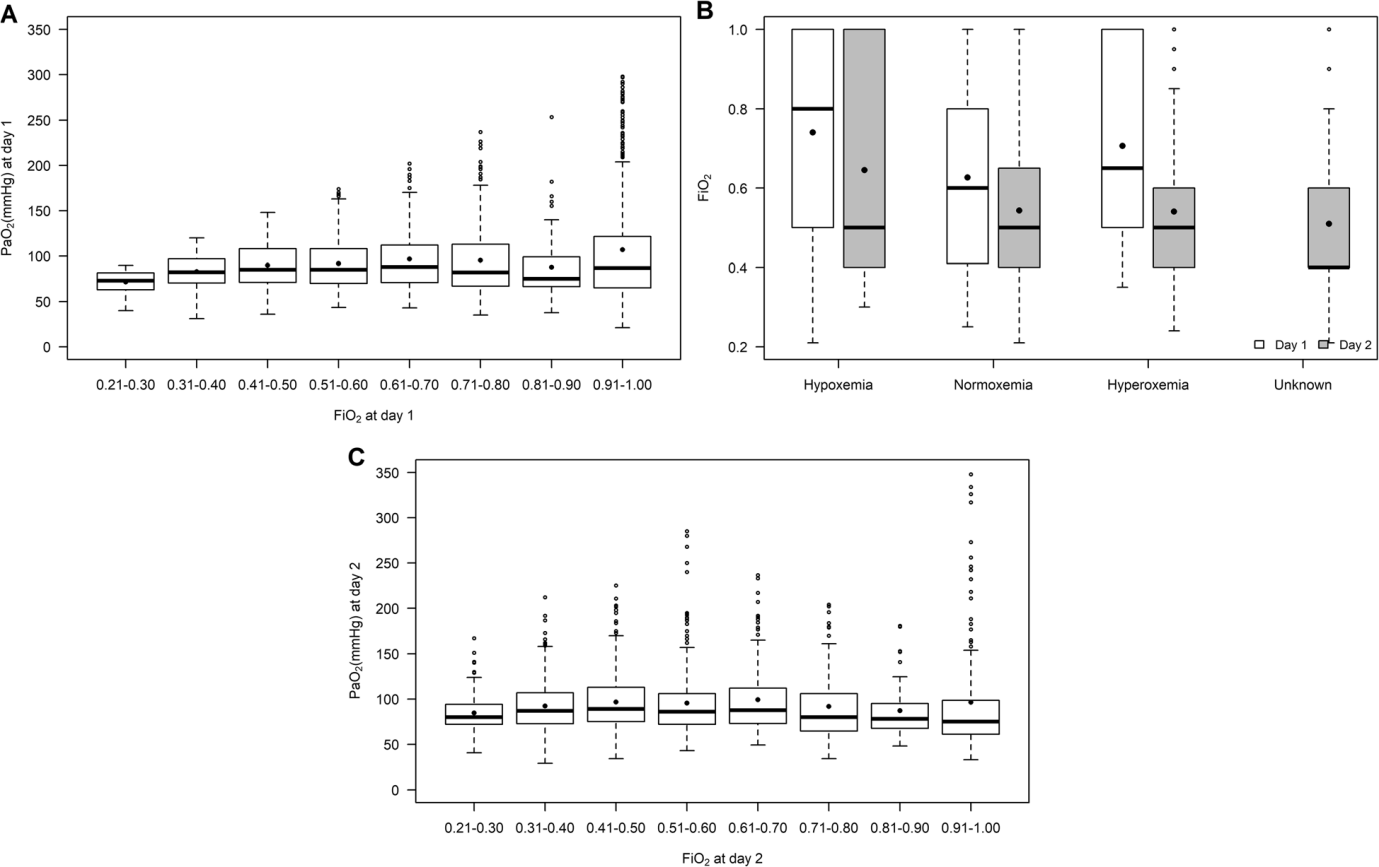
Use of inspired oxygen in patients on days 1 and 2 of ARDS. **a** A box plot of PaO_2_ at each decile of FIO_2_ uses on day 1 of ARDS. **b** A box plot of FIO_2_ used on day 1 and 2 of ARDS in the study population classified by PaO_2_ on day 2 (hypoxemia, normoxemia, hyperoxemia, and unknown). **c** A box plot of PaO_2_ at each decile of FIO_2_ used on day 2 of ARDS

FIO_2_ use varied widely across the spectrum of PaO_2_ on day 1 of ARDS (Fig. 2c). In patients that received a FIO_2_ greater than 0.9 (459 patients), 11% had systemic hypoxemia, while 38% had hyperoxemia (Fig. 2c). Median PaO_2_ was similar across deciles of FIO_2_ (Fig. 3a). On day 2 of ARDS, the proportions of patients receiving higher FIO_2_ decreased, although around one third of patients were hyperoxemic at each decile of FIO_2_ (Figs. 2d and 3b, c). In contrast, 40% (57/131) of patients with hypoxemia on day 1 received a FIO_2_ of 0.5 or less. Median FIO_2_ decreased between day 1 and day 2 in patients with hyperoxemia, normoxemia, and hypoxemia (Fig. 3b), although median PaO_2_ remained similar across deciles of FIO_2_ on day 2 (Fig. 3c).

Excess oxygen use was seen in 400 patients, comprising 66% of all patients with hyperoxemia, on day 1 of ARDS (Table 1). In 315 patients (79%), excess oxygen use was transient, while in 85 (21%) patients, excess oxygen use was also seen on day 2 of ARDS. In multivariable analysis, factors independently associated with excess oxygen use included lower PaO_2_/FIO_2_ ratio, higher PEEP, higher tidal volume, and chronic renal failure (Table 2).

### Hyperoxemia, excess oxygen use, and outcome

**Fig. 4:**
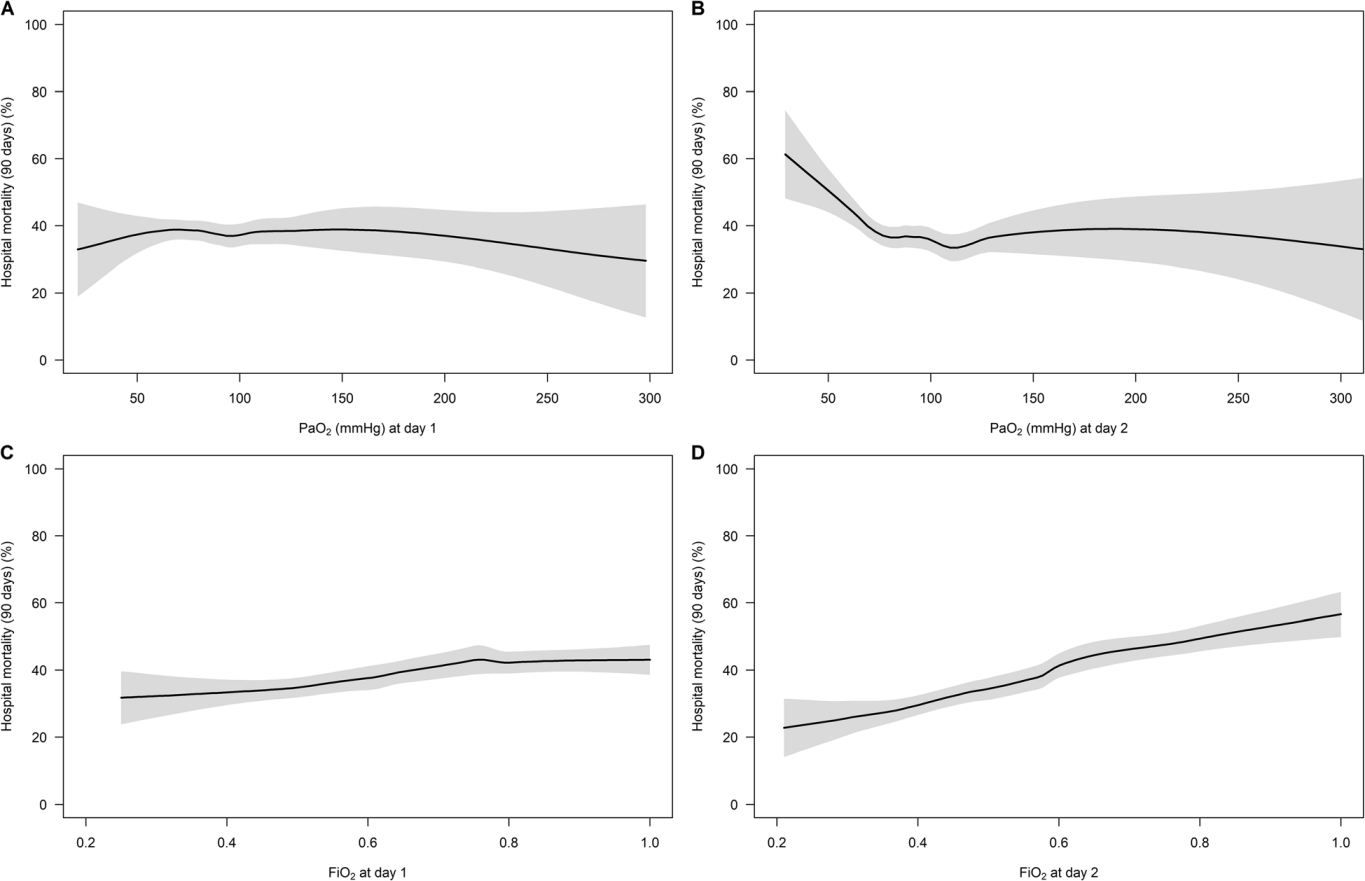
Relationship between oxygen and outcome in patients with ARDS. **a** A locally estimated scatterplot smoothing (LOESS) of the relationship between PaO_2_ on day 1 of ARDS and mortality risk. **b** A LOESS of the relationship between PaO_2_ use on day 2 of ARDS and mortality risk. **c** A LOESS of the relationship between FIO_2_ use on day 1 in non-hypoxemic patients with ARDS and mortality risk. **d** A LOESS of the relationship between FIO_2_ use on day 2 in non-hypoxemic patients with ARDS and mortality risk. Note: LOESS uses a bandwidth 2/3 and 1 degree of polynomial regression

On day 1, LOESS demonstrated the relationship between unadjusted mortality risk and PaO_2_ was relatively flat over the range of PaO2 (Fig. 4a). On day 2, the unadjusted risk of hospital mortality increased in patients with systemic hypoxemia (Fig. 4b). LOESS in non-hypoxemic patients demonstrated that unadjusted mortality risk increased with increasing FIO_2_ on both days 1 and 2 (Fig. 4c, d).

Multivariate analyses found no independent association between day 1 systemic oxygen tension or inspired oxygen concentration and outcome, in either the full study population or in the subset of patients with hyperoxemia (Table 3).

**Table 3:** Factors associated with hospital mortality in study population (*n* = 2005) and in patients with hyperoxemia at day 1 (*n* = 607)

Factor	Odds ratio (95% confidence interval)	*p* value
**Study population** (***n*** **= 2005**)—**model on 1360 patients**		
Age (year)	1.022 (1.015; 1.030)	<.0001
BMI (kg/m^2^)	0.978 (0.962; 0.995)	0.0098
SOFA score—cardiovascular	1.182 (1.096; 1.275)	<.0001
SOFA score—respiratory	1.405 (1.182; 1.669)	0.0001
SOFA score—renal	1.209 (1.087; 1.344)	0.0005
SOFA score—central nervous system	1.147 (1.061; 1.240)	0.0006
Active/hematologic neoplasm or immunosuppression (ref. no.)	2.248 (1.697; 2.978)	<.0001
Chronic liver failure (ref. no.)	4.315 (2.184; 8.523)	<.0001
PIP (cmH_2_O)	1.030 (1.013; 1.046)	0.0003
Invasive mechanical ventilation (ref. no.)	0.497 (0.339; 0.729)	0.0004
Bicarbonate (mmol/L)	0.979 (0.960; 0.997)	0.0240
**Patients with P**_**a**_**O**_**2**_**> 100 mmHg** (***n*** **= 607**)—**model on 530 patients**		
Age (year)	1.031 (1.018; 1.044)	<.0001
SOFA score —renal	1.362 (1.152; 1.610)	0.0003
SOFA score—cardiovascular	1.205 (1.073; 1.352)	0.0016
Active/hematologic neoplasm or immunosuppression (ref. no)	1.828 (1.186; 2.819)	0.0063
Chronic liver failure (ref. no.)	4.091 (1.256; 13.328)	0.0194
Total respiratory rate (breath/min)	1.043 (1.015; 1.072)	0.0027
Bicarbonate (mmol/L)	0.958 (0.924; 0.994)	0.0210

*Abbreviations*: *BMI* body mass index, *PIP* peak inspiratory pressure, *SOFA* sepsis-related organ failure assessment

**Fig. 5:**
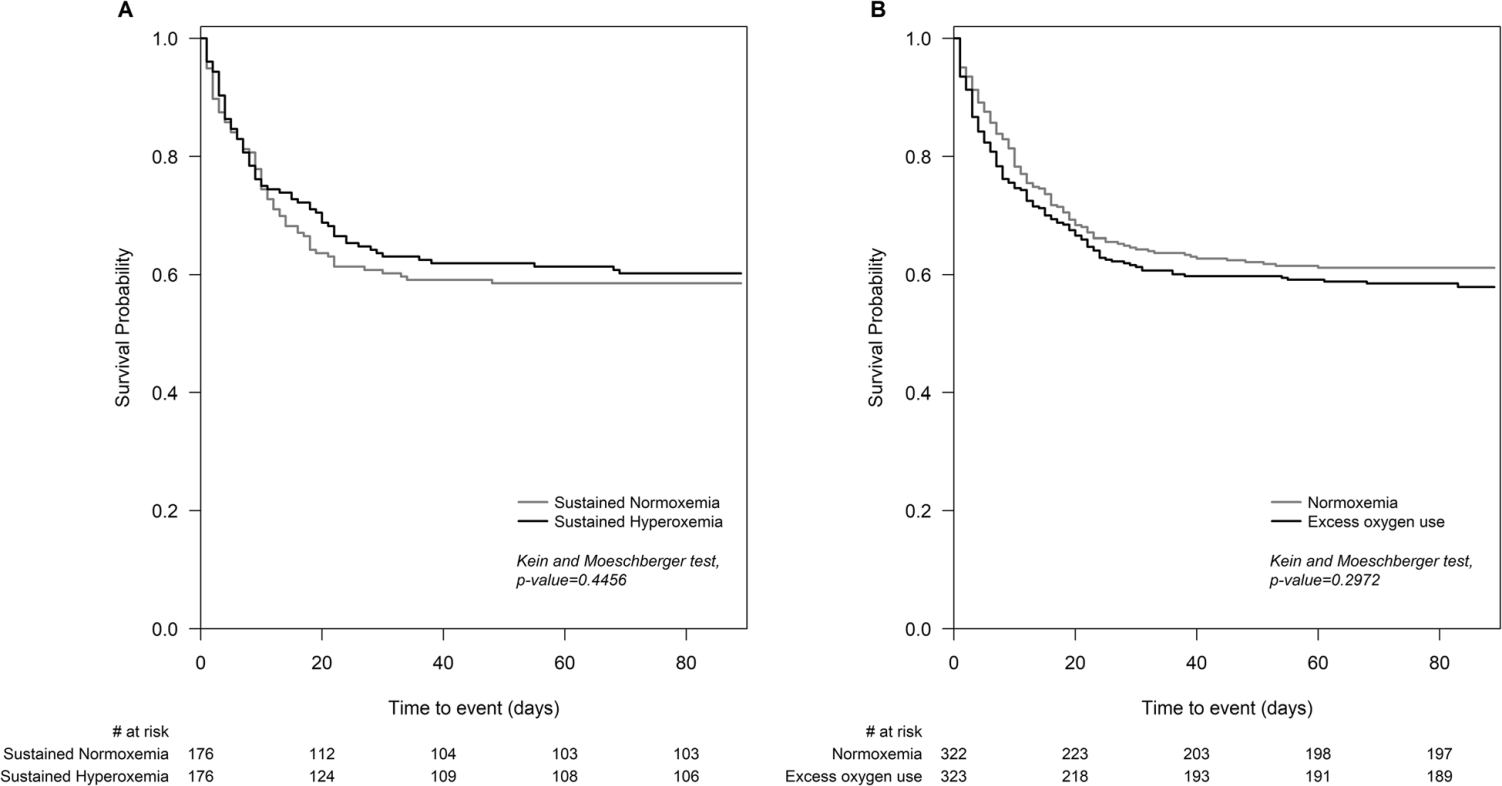
Kaplan-Meier curves for hospital survival in matched samples. **a** Survival probability in matched sample (*n* = 448) of patients with sustained normoxemia and with sustained hyperoxemia. **b** Survival probability in matched sample (*n* = 646) of patients with normoxemia and with excess oxygen use at day 1. Notes: (1) Normoxemia is defined as 55 mmHg ≤ PaO_2_ ≤ 100 mmHg on day 1 of ARDS, sustained normoxemia defined as normoxemia on day 1 and 2 of ARDS, sustained hyperoxemia defined as PaO_2_ > 100 mmHg on day 1 and 2 of ARDS, and excess oxygen use defined as PaO_2_ > 100 mmHg and FIO_2_ ≥ 0.60 on day 1 of ARDS. (2) Mortality is defined as mortality at hospital discharge or at 90 days, whichever event occurred first. We assumed that patients discharged alive from the hospital before 90 days were alive on day 90. (3) The number of patients at risk reported at the bottom of the figure is referred to as the end of the corresponding day

In a propensity-matched analysis (*n* = 448), no outcome differences were found in patients with sustained hyperoxemia compared to matched sustained normoxemia patients (Fig. 5a; eTable 3). Similarly, mortality in patients with hyperoxemia and excess oxygen use (42%) was not different to that in patients with normoxemia (39%, *P* = 0.47) in a propensity-matched sample (*n* = 666) (Fig. 5b; eTable 4).

## Discussion

Our findings demonstrate that hyperoxemia and excess FIO_2_ use was prevalent in patients with early ARDS in patients enrolled in the LUNG SAFE cohort. Hyperoxemia occurred in 30% of patients, while two thirds of these patients received excess oxygen therapy. While a similar proportion of patients was hyperoxemic on day 2 of ARDS, higher FIO_2_ use did decrease. Consequently, most day 2 hyperoxemia was seen in patients at lower FIO_2_, in whom gas exchange was improving. In the majority of patients, both hyperoxemia and excess oxygen use were transient, although sustained hyperoxemia occurred in 12% of patients. Higher FIO_2_ use was independently associated with the risk of hyperoxemia, illustrating the need for close attention to oxygen use to reduce this risk. We found no relationship between the degree and duration of hyperoxemia or of excessive oxygen use, and outcome in early ARDS, in this patient cohort.

### Oxygen use in ARDS

The optimal use of oxygen in patients with ARDS remains unclear. While guidelines recommend the use of supplemental oxygen during acute hypoxemia [38], specific therapeutic goals in terms of PaO_2_ or SpO_2_ are lacking. The ARDS Network targeted a PaO_2_ of 55–80 mmHg in the ARMA trial of patients with ARDS [39]. The British Thoracic Society suggested a target SpO_2_ of 94–98% in acutely ill patients who are not at risk of hypercapnic respiratory failure (only Grade D recommendation) [40, 41].

Tissue hypoxia directly causes cellular death, leading to organ failure and increased mortality in ICU patients. In contrast, high oxygen concentrations may be directly toxic to the lung via mechanisms that remain poorly characterized but may include alveolar-capillary “leak” and fibrogenesis [42, 43], arterial vasoconstriction [18, 19], and the production of reactive oxygen species with consequent proinflammatory and cytotoxic effects [20–22]. Consequently, clinicians are faced with the task of titrating the amount of oxygen delivered to avoid both hypoxemia and hyperoxemia. Prior studies show that clinicians appear to use higher FIO_2_ than is necessary in the critically ill [3]. While the reasons are unclear, potential explanations include concerns over the need to avoid tissue hypoxia, [4, 5] a desire to provide a “buffer” should a clinical deterioration occur, or because the consequences of hyperoxia are considered less severe than hypoxia.

### Hyperoxemia in ARDS

In this study, hyperoxemia was seen on day 1 in a third of ARDS patients enrolled in the LUNG SAFE study. The fact that hyperoxemia was more prevalent than hypoxemia in patients immediately following the onset of ARDS, might seem surprising given that ARDS is a syndrome defined by impaired gas exchange but presumably reflects the effectiveness of ventilatory support and oxygen therapy. Of interest, hyperoxemia was associated with lower SOFA cardiovascular scores, suggesting that clinicians were not permitting hyperoxemia as a “buffer” in patients with shock. In this patient cohort, hyperoxemia was relatively transient in the majority of patients in early ARDS.

A minority of patients had sustained hyperoxemia in this cohort. Interestingly, day 2 median FIO_2_ was the same in patients with sustained hyperoxemia and normoxemia, while P/F ratio was substantially higher in the hyperoxemic patients. These findings suggest that sustained hyperoxemia in these patients is a function of rapidly improving gas exchange rather than excess oxygen use. Sustained hyperoxemia did not have a demonstrable impact on patient mortality. In the matched propensity score analysis, outcomes in patients with sustained hyperoxemia were comparable to that seen in normoxemic patients.

These findings contrast with prior findings regarding hyperoxemia in other critically ill cohorts. However, an important difference between these studies and the current study relates to the severity of hyperoxemia. De Jonge and colleagues reported an association between early hyperoxemia and outcome in patients with acute respiratory failure in the Netherlands [44]. However, this association was only seen in patients with relatively severe hyperoxemia (PaO_2_ > 123 mmHg; uncommon in our cohort) and only on day 1 of ICU admission, while there was no adverse association between hyperoxia over the entire ICU stay and patient outcome. The potential for harm from hyperoxia in the critically ill appears to be enhanced with greater severity and “dose” of hyperoxemia [45]. In fact, in critically ill patient groups where lung function was relatively preserved, such as patients post cardiac arrest, harm was mainly associated with systemic oxygen tensions over 300 mmHg [13]. Greater degrees of hyperoxemia were likely in both the study by Girardis et al. [24] and in the HYPERS2S trial [23] of “induced” systemic hyperoxemia in patients with sepsis. Our study was focused solely on patients with ARDS, where due to their impaired gas exchange, they cannot attain this severity of systemic hyperoxia.

### Oxygen use in ARDS

High inspired oxygen use was frequent in patients on day 1 of ARDS, with two thirds of patients with systemic hyperoxia receiving at least 60% oxygen in day 1—which we termed “excess oxygen use” on the basis that these patients could safely have had their FIO_2_ reduced while maintaining normoxemia. Of importance, high FIO_2_ use was frequently transient, with a marked decrease in higher inspired oxygen concentration use on day 2. Nevertheless, at each decile of FIO_2_, approximately one third of patients were hyperoxemic, suggesting the potential existed to further reduce oxygen use. Of interest, there was an association between excess oxygen use and the use of higher tidal volumes.

Our unadjusted analyses suggested an association between higher FIO_2_ and poorer outcome. However, in multivariate analyses, which accounted for lung injury severity, we found no independent association between high FIO_2_ use and patient outcome. Propensity-matched analyses in patients excess FIO_2_ confirmed no difference in mortality compared to normoxemic patients.

Our findings do not support prior concerns [24] raised regarding the use of higher FIO_2_ in patients with ARDS that are not hypoxemic. This finding also contrasts with the analysis of patients in the ARDS Network trials that found that the cumulative duration of “above target” oxygen exposure (FIO_2_ above 0.5 in ARDS patients while PaO_2_ was > 80 mmHg) was associated with mortality [27]. While the reasons for the divergent findings are unclear, potential explanations include the fact that our analysis concentrated on early ARDS, the fact that high FIO_2_ use was transient in most patients in our cohort, and the fact that this analysis may have been better adjusted for the impact of lung injury severity.

### Limitations

This study has several limitations. The non-linearity of *P*/*F* ratio at different FIO_2_ [46] makes it difficult to predict the effect of FIO_2_ on PaO_2_/FIO_2_, especially when matching patients with mild ARDS. While we have adjusted our analyses to account for known measured confounders, the possibility remains that some of our findings may arise from unmeasured or residual confounding. Moreover, we cannot make causal inferences for any associations seen, given the observational nature of our study. Our dataset comprises daily arterial blood gas and FIO_2_ data, taken at a standardized time each morning. It is possible that these data do not properly reflect the spectrum of FIO_2_ use and PaO_2_ data over the course of that day. Given this, in the hyperoxemia analyses, we focused on patients that were hyperoxemic on both days 1 and 2 of ARDS. There are no single accepted definitions for hyperoxemia, hypoxemia, or excess oxygen use, so our definitions are of necessity arbitrary, and other definitions have been used in other analyses. This could partly explain any divergence in findings across these studies. Lastly, our assumption that inpatients at day 90 survived to hospital discharge is a further limitation.

## Conclusions

Our findings demonstrate that hyperoxemia and high fractional inspired oxygen use is prevalent in patients with early ARDS in patients enrolled in the LUNG SAFE cohort. Higher FIO_2_ use decreased from day 1 to day 2 of ARDS, with most day 2 hyperoxemia seen in patients at lower FIO_2_, in whom gas exchange was improving. Reassuringly, we found no relationship between hyperoxemia or excessive oxygen use and patient outcome in this cohort.

## Supplementary information

**Additional file 1. **Online Methodology and eTables. Expanded Methods and Materials. **eTable 1**: Comorbidities and risk factors in study population (*n* = 2005), stratified by arterial oxygenation on day 1. **eTable 2**. Characteristics of patients with sustained normoxemia and sustained hyperoxemia. **eTable 3**: Characteristics at ARDS onset and clinical outcomes in matched sample (*n* = 354) of patients with sustained normoxemia and with sustained hyperoxemia. **eTable 4**. Characteristics at ARDS onset and clinical outcomes in matched sample (*n* = 646) of patients with normoxemia and with excess oxygen use at day 1.

## Data Availability

Available upon request

## Abbreviations

ARDS: Acute respiratory distress syndrome
ICUs: Intensive care units
LUNG SAFE: Large Observational Study to Understand the Global Impact of Severe Acute Respiratory Failure
ESICM: European Society of Intensive Care Medicine
PaO_2_: Arterial oxygen tension
FIO_2_: Fraction of inspired oxygen
MV: Invasive mechanical ventilation
ANOVA: Analysis of variance
LOESS: Locally estimated scatterplot smoothing
OR: Odds ratio
CI: Confidence interval
SOFA: Sepsis-related organ failure assessment
