# Supplementary material for: Hyperoxemia and excess oxygen use in early acute respiratory distress syndrome: insights from the LUNG SAFE study
Source: Crit Care. 2020 Mar 31;24:125. doi: 10.1186/s13054-020-2826-6 (PMC7110678; doi:10.1186/s13054-020-2826-6)
Supplement: Supplementary file 1 — Additional file 1. Online Methodology and eTables. Expanded Methods and Materials. eTable 1: Comorbidities and risk factors in study population (n = 2005), stratified by arterial oxygenation on day 1. eTable 2. Characteristics of patients with sustained normoxemia and sustained hyperoxemia. eTable 3: Characteristics at ARDS onset and clinical outcomes in matched sample (n = 354) of patients with sustained normoxemia and with sustained hyperoxemia. eTable 4. Characteristics at ARDS onset and clinical outcomes in matched sample (n = 646) of patients with normoxemia and with excess oxygen use at day 1. [file 13054_2020_2826_MOESM1_ESM.docx]

**Online Methodology end eTables**

**Title:** Hyperoxemia and excess oxygen use in patients with early Acute Respiratory Distress Syndrome: Insights from the LUNG SAFE study

Fabiana Madotto, PhD, Emanuele Rezoagli MD, PhD, Tài Pham, MD, PhD, Marcello Schmidt, MD, Bairbre McNicholas MD, PhD, Alessandro Protti, MD, PhD, Rakshit Panwar, MD, FCICM^,^ Giacomo Bellani, MD, PhD, Eddy Fan, MD, PhD, Frank van Haren MD, Laurent Brochard, MD, PhD, John G. Laffey, MD, MA, On behalf of the LUNG SAFE Investigators and the ESICM Trials Group

**Methods and Materials**

The detailed methods and protocol for the LUNG SAFE study have previously been published elsewhere [1]. In brief, LUNG SAFE was an international, multicenter, prospective cohort study, with a 4-week enrollment window in the winter season in both hemispheres (February-March in the northern hemisphere and June-August 2014 in the southern hemisphere) [1]. The study, supported by the European Society of Intensive Care Medicine (ESICM), was endorsed by multiple national societies/networks (***Appendix 1***). All participating Intensive Care Units (ICUs) obtained ethics committee approval, and either patient consent or ethics committee waiver of consent. National coordinators (***Appendix 1***) and site investigators (***Appendix 1***) were responsible for obtaining ethics committee approval and for ensuring data integrity and validity. Data were collected by means of an electronic case report form (eCRF, Clinfile®, Paris, France) and data quality was subsequently verified on the database and investigators were queried in regard to outlier or inconsistent data.

***Patients, Study Design and Data Collection***

LUNG SAFE study enrolled all patients admitted to participating ICUs within the 4-week enrollment window and receiving invasive or noninvasive mechanical ventilation (MV). Exclusion criteria were: age<16 years or inability to obtain informed consent (where required). Following enrollment, patients were evaluated daily for acute hypoxemic respiratory failure (AHRF), defined as the concurrent presence of: (1) PaO_2_/FiO_2_≤300 mmHg; (2) new pulmonary parenchymal abnormalities on chest X-Ray or computed tomography; and (3) ventilatory support with continuous positive airway pressure (CPAP) or expiratory positive airway pressure (EPAP) or positive end expiratory pressure (PEEP) ≥5 cmH_2_O. If this condition was present, patients were classified as having acute respiratory distress syndrome (ARDS) based on whether or not they fulfilled all of the Berlin criteria rather than by clinician determination, as previously described [1].

Given the study focus on early hyperoxemia and use excess oxygen use, we restricted the study population to patients that fulfilled ARDS criteria within 48 hours of ICU admission, and who remained in ICU for at least 2 days from ARDS onset. Patients transferred from other ICUs after 2 days, patients that developed ARDS later in their ICU stay, and patients that received early ECMO were excluded [***Figure 1***].

Data on arterial blood gases, type of ventilatory support with relative settings and Sequential Organ Failure Assessment (SOFA) score were collected on selected days during the ICU stay. Data were collected once per day: if more than one value was available during the day, investigator were asked to record data collected as close as possible to 10 AM. Data on ventilatory settings were recorded simultaneously with arterial blood gas. Decisions to withhold or withdraw life sustaining treatments during the ICU stay and the time at which this decision was taken were recorded (all-time treatment limitations). ICU and hospital survival were collected at the time of discharge, censored at 90 days after enrollment (whichever occurred earlier).

***Data Definitions***

For the purposes of this analysis the following definitions were applied on day 1 and on day 2 of ARDS: hypoxemia (PaO_2_ < 55 mmHg); normoxemia (PaO_2_ 55 – 100 mmHg), and hyperoxemia (PaO_2_ > 100 mmHg). Excess oxygen us was defined as the use of FiO_2_ ≥ 0.6 in patients with hyperoxemia (PaO_2_ > 100 mmHg). Patients with hyperoxemia on days 1 and 2 of ARDS were considered to have sustained hyperoxemia. Analogously, we also defined patients with sustained hypoxemia and sustained normoxemia.

Because bicarbonate concentrations were not collected in LUNG SAFE study, we derived them from variables collected (pH and partial pressure of carbon dioxide) using Henderson-Hasselbach equation. We also derived: 1) dynamic compliance (ml/cmH_2_O) as the ratio between tidal volume and the difference between peak inspiratory pressure and PEEP; 2) body mass index (BMI) as the ratio between weight (kilograms) and the square of the body height (meters). Moreover, driving pressure was defined as plateau pressure minus PEEP. We analyzed plateau and driving pressure in patients where plateau pressure was measured and in whom there was no evidence of spontaneous ventilation (i.e. when set and measured respiratory rates were equal). All modes other than volume and pressure control modes were considered to permit spontaneous breathing.

The duration of invasive MV was calculated as the number of days between the date of intubation and extubation in ICU (or death, if patient died under invasive MV). Similarly, invasive ventilator-free days were calculated as the number of days from weaning from invasive MV to day 28 and for patients who died before weaning, we considered to have a ventilator-free-day value of 0.

Patient survival was evaluated at hospital discharge, or at day 90, whichever occurred first. Our other data definitions have been previously reported [1-4].

***Data Management and Statistical analyses***

Descriptive statistics included proportions for categorical and mean (standard deviation) or median (interquartile range) for continuous variables. No assumptions were made for missing data. To assess differences among three groups (systemic hypoxemia, normoxemia, and hyperoxemia) we performed chi-squared test (or Fisher exact test) for discrete variables, analysis of variance (ANOVA) (or Kruskal-Wallis test) for continuous variables. Bonferroni correction was applied to determine significance in the setting of multiple comparisons. Chi-square test (or Fisher exact test), Student’s T-test (or Wilcoxon Mann Whitney test) were used to assess differences between groups (i.e. sustained hyperoxemia and sustained normoxemia) in discrete and continuous distributions of parameters, respectively.

To investigate relationship between hospital mortality and PaO_2_ and FiO_2_ levels observed during day 1 and day 2 from ARDS onset, we used locally estimated scatterplot smoothing (LOESS) with a bandwidth of 2/3 and 1 degree of polynomial regression.

Multivariable logistic regression models were used to evaluate factors associated with: (a) the presence of hyperoxemia; (b) the excess of oxygen use; and (c) mortality.

In each regression model, the independent predictors (demographic characteristics and clinical parameters measured at the first day of ARDS) were identified through a stepwise regression approach. This approach combines forward and backward selection methods in an iterative procedure (with a significance level of 0.05 both for entry and retention) to select predictors in the final multivariable model. The list of possible predictors included demographic characteristics (age, sex), body mass index, comorbidities (presence of heart failure, diabetes mellitus, chronic liver failure, chronic renal failure, chronic obstructive pulmonary disease or home ventilation, active neoplasm of hematologic neoplasm or immunosuppression), presence of at least one ARDS risk factors, bicarbonates concentration, management factors (presence of invasive mechanical ventilation, tidal volume, PEEP, PIP, total respiratory rate, standardized minute ventilation). Moreover, we considered FiO_2_ and five (on six) components of SOFA score (cardiovascular, liver, coagulation, renal, central nervous system) as possible predictors for presence of hypoxemia; while PaO_2_/FiO_2_ ratio and non-respiratory SOFA score (adjusted for missing data) were considered as possible predictors for excess of oxygen use. Regarding the multivariable logistic model on mortality, all components of SOFA score, FiO_2_ and PaO_2_ were included in the list of possible predictors. Models’ results were reported as odds ratio (OR) with 95% confidence interval (CI).

Propensity score matching method was applied to evaluate the possible impact of sustained hyperoxemia versus sustained normoxemia on main outcomes (mortality, ventilation free days and duration of MV) in patients with ARDS. In detail, patients were matched (1:1 match without replacement), using a caliper of 0.2 standard deviation of the logit of the propensity score and the similarity of the matched groups was assessed by the standardized differences of each independent variable used in the propensity score estimation. The balance in measured variables between groups has been assessed using standardized difference and a value of less than 0.10 likely denoted a negligible imbalance. Statistical significance of the difference in the ventilation free days and in the duration of MV was evaluated with Wilcoxon signed-rank test, while for difference in proportions of deaths we applied McNemar’s test. Propensity score matching method was also used to examine the possible impact of excess use of oxygen on main outcomes. Survival probability in these matched groups was estimated using the Kaplan-Meier approach and assuming that patients discharged alive from hospital before 90 days were alive on day 90. Statistical difference between survival curves was assessed through Kein and Moeschberger test.

All p-values were two-sided, with p-values <0.05 considered as statistically significant.

Statistical analyses were performed with R, version 3.5.2. (R Project for Statistical Computing, <http://www.R-project.org>) and SAS software, version 9.4 (SAS Institute, Cary, NC, USA).

**References**

1. Bellani G, Laffey JG, Pham T, Fan F, Brochard L, Esteban A, Gattinoni L, van Haren F, Larsson A, McAuley DF *et al*: **Epidemiology, patterns of care, and mortality for patients with acute respiratory distress syndrome in intensive care units in 50 countries**. *JAMA* 2016, **315**(8):788-800.

2. Bellani G, Laffey JG, Pham T, Madotto F, Fan E, Brochard L, Esteban A, Gattinoni L, Bumbasirevic V, Piquilloud L *et al*: **Non-invasive Ventilation of Patients with Acute Respiratory Distress Syndrome: Insights from the LUNG SAFE Study**. *Am J Respir Crit Care Med* 2017, **195**(1):67-77.

3. Laffey JG, Bellani G, Pham T, Fan E, Madotto F, Bajwa EK, Brochard L, Clarkson K, Esteban A, Gattinoni L *et al*: **Potentially modifiable factors contributing to outcome from acute respiratory distress syndrome: the LUNG SAFE study**. *Intensive Care Med* 2016, **42**(12):1865-1876.

4. McNicholas BA, Madotto F, Pham T, Rezoagli E, Masterson CH, Horie S, Bellani G, Brochard L, Laffey JG, Group LSIatET: **Demographics, management and outcome of women and men with Acute Respiratory Distress Syndrome in the LUNG SAFE prospective cohort study**. *Eur Respir J* 2019.

**eTable 1:** Comorbidities and risk factors in study population (n=2,005), stratified by arterial oxygenation on day 1.

| **Parameter** | **Hypoxemia**  (*PaO_2_ < 55 mmHg*) | **Normoxemia**  (*55 mmHg ≤ PaO_2_ ≤ 100 mmHg*) | **Hyperoxemia**  (*PaO_2_ > 100 mmHg*) | p-value  (*Comparison among groups*) |
| --- | --- | --- | --- | --- |
| **Comorbidities, n (%)** |  |  |  |  |
| Heart failure | 18 (13.74) | 124 (9.79) | 80 (13.18) | 0.0547 |
| Diabetes mellitus | 23 (17.56) | 284 (22.42) | 145 (23.89) | 0.2857 |
| Chronic renal failure | 17 (12.98) | 120 (9.47) | 82 (13.51)^†^ | 0.0237 |
| Chronic liver failure | 5 (3.82) | 50 (3.95) | 16 (2.64) | 0.3509 |
| COPD or home ventilation | 30 (22.90) | 314 (24.78) | 134 (22.08) | 0.4222 |
| COPD | 29 (22.14) | 302 (23.84) | 129 (21.25) | 0.4491 |
| Home ventilation | 4 (3.05) | 31 (2.45) | 11 (1.81) | 0.5777 |
| Active/hematologic neoplasm or immunosuppression | 28 (21.37) | 257 (20.28) | 149 (24.55) | 0.1106 |
| Active neoplasm | 8 (6.11) | 109 (8.60) | 67 (11.04) | 0.1052 |
| Hematologic neoplasm | 7 (5.34) | 54 (4.26) | 27 (4.45) | 0.8444 |
| Immunosuppression | 22 (16.79) | 157 (12.39) | 80 (13.18) | 0.3502 |
| **ARDS risk factors, n (%)** |  |  |  |  |
| Pneumonia | 93 (70.99) | 779 (61.48) | 361 (59.47)* | 0.0488 |
| Aspiration of gastric contents | 20 (15.27) | 190 (15.00) | 99 (16.31) | 0.7613 |
| Inhalation injury | 4 (3.05) | 30 (2.37) | 16 (2.64) | 0.8599 |
| Pulmonary contusion | 4 (3.05) | 31 (2.45) | 19 (3.13) | 0.6701 |
| Pulmonary vasculitis | 0 (0.00) | 5 (0.39) | 3 (0.49) | 0.8365 |
| Drowning | 0 (0.00) | 0 (0.00) | 0 (0.00) | - |
| Non-pulmonary sepsis | 19 (14.50) | 202 (15.94) | 90 (14.83) | 0.7793 |
| Trauma | 4 (3.05) | 39 (3.08) | 25 (4.12) | 0.4954 |
| Pancreatitis | 0 (0.00) | 30 (2.37) | 11 (1.81) | 0.1688 |
| Severe burns | 0 (0.00) | 1 (0.08) | 3 (0.49) | 0.2203 |
| Non-cardiogenic shock | 10 (7.63) | 94 (7.42) | 43 (7.08) | 0.9576 |
| Drug overdose | 2 (1.53) | 26 (2.05) | 9 (1.48) | 0.6659 |
| TRALI | 2 (1.53) | 56 (4.42) | 22 (3.62) | 0.2348 |
| Other | 1 (0.76) | 24 (1.89) | 24 (3.95)^†^ | 0.0113 |

*Abbreviations: ARDS: acute respiratory distress syndrome; COPD: chronic obstructive pulmonary disease; PaO_2_: arterial oxygen partial pressure; TRALI: transfusion related acute lung injury.*

** p-value < 0.05 (Bonferroni’s correction), comparison with “Hypoxemia” group.*

*† p-value < 0.05 (Bonferroni’s correction), comparison with “Normoxemia” group.*

**eTable 2.** Characteristics of patients with sustained normoxemia and sustained hyperoxemia.

| **Parameter** | **Sustained**  **normoxemia** | **Sustained**  **hyperoxemia** | p-value |
| --- | --- | --- | --- |
| N | 810 | 250 |  |
| Male, n (%) | 513 (63.33) | 156 (62.40) | 0.7892 |
| Age (years), mean ± SD | 61.94 ± 16.54 | 61.53 ± 17.21 | 0.8702 |
| BMI (kg/m^2^), mean ± SD | 27.87 ± 8.34 | 26.15 ± 5.86 | 0.0152 |
| **Comorbidities, n (%)** |  |  |  |
| Heart failure | 76 (9.38) | 36 (14.40) | 0.0241 |
| Diabetes mellitus | 187 (23.09) | 51 (20.40) | 0.3735 |
| Chronic renal failure | 77 (9.51) | 33 (13.20) | 0.0941 |
| Chronic liver failure | 32 (3.95) | 7 (2.80) | 0.3982 |
| COPD or home ventilation | 209 (25.80) | 47 (18.80) | 0.0237 |
| COPD | 201 (24.81) | 44 (17.60) | 0.0180 |
| Home ventilation | 20 (2.47) | 3 (1.20) | 0.2286 |
| Active/hematologic neoplasm or immunosuppression | 166 (20.49) | 67 (26.80) | 0.0353 |
| Active neoplasm | 73 (9.01) | 34 (13.60) | 0.0353 |
| Hematologic neoplasm | 33 (4.07) | 9 (3.60) | 0.7369 |
| Immunosuppression | 102 (12.59) | 37 (14.80) | 0.3661 |
| **ARDS risk factors, n (%)** |  |  | 0.4563 |
| None | 64 (7.90) | 19 (7.60) |  |
| Only non-pulmonary | 143 (17.65) | 44 (17.60) |  |
| Only pulmonary | 499 (61.60) | 145 (58.00) |  |
| Both | 104 (12.84) | 42 (16.80) |  |
| **Illness severity** |  |  |  |
| **Day 1** |  |  |  |
| PaO_2_ (mmHg), mean ± SD | 76.39 ± 11.75 | 140.50 ± 42.91 | <.0001 |
| PaO_2_/FiO_2_ (mmHg), mean ± SD | 140.02 ± 55.76 | 208.78 ± 54.47 | <.0001 |
| ARDS severity, n (%) |  |  | <.0001 |
| Mild | 123 (15.19) | 138 (55.20) | <.0001 |
| Moderate | 443 (54.69) | 112 (44.80) | 0.0062 |
| Severe | 244 (30.12) | 0 (0.00) | <,0001 |
| PaCO_2_ (mmHg), mean ± SD | 46.87 ± 15.69 | 43.86 ± 12.84 | 0.0302 |
| pH, mean ± SD | 7.33 ± 0.12 | 7.32 ± 0.13 | 0.9781 |
| Bicarbonate (mmol/L), mean ± SD | 23.61 ± 6.66 | 22.00 ± 5.99 | 0.0012 |
| Base excess (mEq/L), mean ± SD | -1.75 ± 6.91 | -3.30 ± 6.59 | 0.0018 |
| Non-respiratory SOFA score adjusted, mean ± SD | 6.04 ± 3.97 | 6.31 ± 4.16 | 0.4226 |
| SOFA score adjusted, mean ± SD | 9.46 ± 4.01 | 8.88 ± 4.08 | 0.0392 |
| Dopamine (µg/kg/min), mean ± SD | 8.42 ± 5.28 | 7.89 ± 6.74 | 0.4458 |
| Dobutamine (µg/kg/min), mean ± SD | 5.42 ± 3.55 | 6.64 ± 4.20 | 0.5455 |
| Noradrenaline (µg/kg/min), mean ± SD | 0.43 ± 0.77 | 0.42 ± 0.68 | 0.4422 |
| Adrenaline (µg/kg/min), mean ± SD | 0.47 ± 0.73 | 0.48 ± 0.68 | 0.5786 |
| **Day 2** |  |  |  |
| PaO_2_ (mmHg), mean ± SD | 77.2 ± 11.6 | 138.0 ± 42.8 | <.0001 |
| PaO_2_/FiO_2_ (mmHg), mean ± SD | 162.48 ± 62.03 | 271.87 ± 81.23 | <.0001 |
| PaCO_2_ (mmHg), mean ± SD | 45.1 ± 14.4 | 40.5 ± 10.5 | <.0001 |
| pH, mean ± SD | 7.36 ± 0.10 | 7.37 ± 0.11 | 0.1905 |
| Bicarbonate (mmol/L), mean ± SD | 24.6 ± 6.7 | 22.7 ± 6.1 | <.0001 |
| Base excess (mEq/L), mean ± SD | 0.4 ± 6.8 | -2.1 ± 6.6 | 0.0004 |
| Non-respiratory SOFA score adjusted, mean ± SD | 6.33 ± 4.34 | 6.29 ± 4.19 | 0.8436 |
| SOFA score adjusted, mean ± SD | 9.56 ± 4.62 | 8.08 ± 4.41 | <.0001 |
| Dopamine (µg/kg/min), mean ± SD | 7.35 ± 3.95 | 6.21 ± 5.75 | 0.0787 |
| Dobutamine (µg/kg/min), mean ± SD | 5.42 ± 4.26 | 7.83 ± 6.52 | 0.1549 |
| Noradrenaline (µg/kg/min), mean ± SD | 0.52 ± 1.03 | 0.64 ± 1.76 | 0.1813 |
| Adrenaline (µg/kg/min), mean ± SD | 1.44 ± 4.23 | 0.83 ± 1.38 | 0.9595 |
| **Management factors** |  |  |  |
| **Day 1** |  |  |  |
| Invasive mechanical ventilation, n (%) | 633 (78.15) | 215 (86.00) | 0.0067 |
| Control mode of ventilation, n (%) | 439 (55.57) | 164 (65.60) | 0.0051 |
| FiO_2_, median (q_1_; q_3_) | 0.60 (0.42 ; 0.80) | 0.65 (0.50 ; 1.00) | <.0001 |
| FiO_2_ ≥ 0.6, n (%) | 427 (52.72) | 170 (68.00) | <.0001 |
| Tidal volume (ml/kg), mean ± SD | 7.8 ± 2.0 | 7.7 ± 1.7 | 0.7187 |
| PEEP (cmH_2_O), mean ± SD | 8.2 ± 3.2 | 7.9 ± 3.1 | 0.0606 |
| PIP (cmH_2_O), mean ± SD | 25.2 ± 8.2 | 25.4 ± 7.9 | 0.9165 |
| Total respiratory rate (breaths/min), mean ± SD | 22.0 ± 7.1 | 20.6 ± 6.9 | 0.0007 |
| Standardized minute ventilation (L/min), mean ± SD | 11.7 ± 5.71 | 10.2 ± 4.5 | <.0001 |
| Patients in whom plateau pressure measured°, n (%) | 180 (22.22) | 88 (35.20) | <.0001 |
| Plateau pressure (cmH_2_O), mean ± SD | 23.3 ± 6.0 | 22.1 ± 5.4 | 0.1535 |
| Driving pressure (cmH_2_O), mean ± SD | 14.4 ± 5.1 | 14.2 ± 5.3 | 0.8329 |
| **Day 2** |  |  |  |
| Invasive mechanical ventilation, n (%) | 631 (77.90) | 231 (84.00) | 0.0306 |
| Control mode of ventilation, n (%) | 374 (51.02) | 143 (55.64) | 0.2021 |
| FiO_2_, median (q_1_; q_3_) | 0.50 (0.40; 0.65) | 0.50 (0.40; 0.60) | 0.8319 |
| FiO_2_ ≥ 0.6, n (%) | 308 (38.26) | 89 (35.74) | 0.4736 |
| Tidal volume (ml/kg), mean ± SD | 7.9 ± 2.0 | 7.8 ± 1.8 | 0.5985 |
| PEEP (cmH_2_O), mean ± SD | 8.5 ± 3.4 | 7.7 ± 3.3 | 0.0002 |
| PIP (cmH_2_O), mean ± SD | 25.2 ± 8.1 | 24.1 ± 7.6 | 0.0395 |
| Total respiratory rate (breaths/min), mean ± SD | 21.5 ± 6.4 | 20.6 ± 6.3 | 0.0211 |
| Standardized minute ventilation (L/min), mean ± SD | 11.2 ± 4.5 | 9.7 ± 3.9 | <.0001 |
| Patients in whom plateau pressure measured°, n (%) | 177 (21.85) | 72 (26.18) | 0.1401 |
| Plateau pressure (cmH_2_O), mean ± SD | 23.3 ± 6.0 | 22.8 ± 4.7 | 0.4529 |
| Driving pressure (cmH_2_O), mean ± SD | 14.2 ± 5.2 | 14.6 ± 5.3 | 0.6919 |
| **Clinical outcomes** |  |  |  |
| Hospital mortality (90 days) | 324 (40.20) | 92 (37.10) | 0.3822 |
| ICU mortality (90 days) | 276 (34.07) | 78 (31.20) | 0.3996 |
| Hospital length of stay (days), median (q_1_; q_3_) |  |  |  |
| All | 16.0 (8.0; 29.0) | 17.0 (9.0; 31.0) | 0.4066 |
| Alive at hospital discharge | 20.0 (13.0; 37.0) | 21.0 (12.0; 38.0) | 0.9699 |
| Ventilation free days (days), median (q_1_; q_3_) |  |  |  |
| All | 10.0 (0.0; 22.0) | 17.0 (0.0; 24.0) | 0.0045 |
| Alive at ICU discharge | 20.0 (13.0; 24.0) | 22.0 (18.0; 26.0) | 0.0009 |
| Duration mechanical ventilation (days), median (q_1_; q_3_) |  |  |  |
| All | 9.0 (4.0; 16.0) | 7.0 (4.0; 13.0) | 0.0088 |
| Alive at ICU discharge | 9.0 (5.0; 16.0) | 7.0 (3.0; 11.0) | 0.0010 |

*Abbreviations: ARDS: acute respiratory distress syndrome; BMI: body mass index; COPD: chronic obstructive pulmonary disease; FiO_2_: fraction of inspired oxygen; ICU: intensive care unit; P_a_O_2_: arterial oxygen partial pressure; P_a_CO_2_: arterial carbon dioxide partial pressure; PEEP: positive end-expiratory pressure; PIP: peak inspiratory pressure; q_1_: first quartile; q_3_: third quartile; SOFA: sepsis-related organ failure assessment; SD: standard deviation.*

*° Plateau pressure and driving pressure values are limited to patients in whom this value was reported and in whom either an assist control mode was used or in whom a mode permitting spontaneous ventilation was used and where the set and total respiratory rates were equal. Patients receiving HFOV or ECMO were also excluded.*

**eTable 3:** Characteristics at ARDS onset and clinical outcomes in matched sample (n=354) of patients with sustained normoxemia and with sustained hyperoxemia.

| **Parameter** | **Sustained Normoxemia**  **(n=177)** | **Sustained Hyperoxemia**  **(n=177)** | Standardize  difference |
| --- | --- | --- | --- |
| Age (years), mean ± SD | 62.73 ± 16.66 | 61.31 ± 17.42 | 0.08 |
| Males, n (%) | 104 (58.76) | 112 (63.28) | 0.09 |
| BMI (kg/m^2^) , mean ± SD | 26.39 ± 5.92 | 26.25 ± 6.20 | 0.02 |
| Chronic diseases, n (%) |  |  |  |
| Heart failure | 26 (14.69) | 22 (12.43) | 0.07 |
| Diabetes mellitus | 40 (22.60) | 36 (20.34) | 0.06 |
| Chronic renal failure | 22 (12.43) | 21 (11.86) | 0.02 |
| Chronic liver failure | 5 (2.82) | 6 (3.39) | 0.03 |
| COPD or home ventilation | 43 (24.29) | 36 (20.34) | 0.10 |
| Active/hematologic neoplasm or immunosuppression | 47 (26.55) | 49 (27.69) | 0.03 |
| At least 1 ARDS risk factor, n (%) | 165 (93.22) | 164 (92.66) | 0.02 |
| Bicarbonate (mmol/L), mean ± SD | 22.3 ± 6.0 | 22.1 ± 5.7 | 0.03 |
| Non-respiratory SOFA score adjusted, mean ± SD | 6.77 ± 3.91 | 6.46 ± 4.31 | 0.04 |
| Invasive mechanical ventilation, n (%) | 160 (90.40) | 155 (87.57) | 0.09 |
| Dynamic compliance (ml/cmH_2_O), mean ± SD | 34.3 ± 24.6 | 33.7 ± 24.7 | 0.03 |
| Total respiratory rate (breaths/min), mean ± SD | 20.5 ± 6.0 | 20.8 ± 7.3 | 0.04 |
| PaO_2_ / FiO_2_ (mmHg), mean ± SD | 196.80 ± 50.94 | 194.63 ± 53.35 | 0.04 |
|  |  |  | p-value |
| **Variables not included in propensity score estimation** |  |  |  |
| FiO_2_, median (q_1_; q_3_) | 0.40 (0.35 ; 0.50) | 0.70 (0.55 ; 1.00) | <.0001 |
| PaO_2_ (mmHg), mean ± SD | 82.3 ± 10.8 | 134.7 ± 36.4 | <.0001 |
| ARDS severity, n (%) |  |  | 0.5424 |
| Mild | 81 (45.76) | 76 (42.94) |  |
| Moderate | 96 (54.24) | 101 (57.06) |  |
| **Clinical Outcomes** |  |  |  |
| Hospital mortality (90 days), n (%) | 66 (37.50) | 70 (40.00) | 0.6683 |
| Ventilation free days (days), median (q_1_; q_3_) | 16.0 (0.0 ; 24.0) | 16.0 (0.0 ; 24.0) | 0.6109 |
| Duration mechanical ventilation (days), median (q_1_; q_3_) | 7.0 (4.0 ; 12.5) | 7.0 (4.0 ; 13.0) | 0.9144 |

*Abbreviations: ARDS: acute respiratory distress syndrome; BMI: body mass index; COPD: chronic obstructive pulmonary disease; FiO_2_: fraction of inspired oxygen; P_a_O_2_: arterial oxygen partial pressure; q_1_: first quartile; q_3_: third quartile; SD: standard deviation; SOFA: sepsis-related organ failure assessment.*

*Notes: 1) Sustained normoxemia defined as 55 mmHg ≤ PaO_2_ ≤ 100 mmHg on day 1 and 2 of ARDS. 2) Sustained hyperoxemia defined as P_a_O_2_ > 100mmHg on day 1 and 2 of ARDS; (3) Patients with severe ARDS at day 1 were excluded from propensity score matching.*

**eTable 4.** Characteristics at ARDS onset and clinical outcomes in matched sample (n=646) of patients with normoxemia and with excess oxygen use at day 1.

| **Parameter** | **Normoxemia**  **(n=323)** | **Excess oxygen use**  **(n=323)** | Standardize  difference |
| --- | --- | --- | --- |
| Age (years), mean ± sd | 62.25 ± 15.82 | 61.44 ± 16.66 | 0.05 |
| Males, n (%) | 199 (61.61) | 200 (61.91) | 0.01 |
| BMI (kg/m^2^) , mean ± SD | 27.10 ± 7.03 | 27.06 ± 6.52 | 0.01 |
| Chronic diseases, n (%) |  |  |  |
| Heart failure | 46 (14.24) | 41 (13.00) | 0.04 |
| Diabetes mellitus | 86 (26.63) | 85 (26.32) | 0.01 |
| Chronic renal failure | 41 (12.69) | 37 (11.46) | 0.04 |
| Chronic liver failure | 8 (2.49) | 9 (2.79) | 0.02 |
| COPD or home ventilation | 86 (26.63) | 76 (23.53) | 0.07 |
| Active/hematologic neoplasm or immunosuppression | 83 (25.70) | 81 (25.08) | 0.01 |
| At least 1 ARDS risk factor, n (%) | 300 (92.88) | 293 (90.71) | 0.08 |
| Bicarbonate (mmol/L), mean ± SD | 22.1 ± 6.1 | 22.1 ± 6.2 | 0.00 |
| Non-respiratory SOFA score adjusted, mean ± SD | 6.49 ± 3.87 | 6.63 ± 4.05 | 0.04 |
| Invasive mechanical ventilation, n (%) | 283 (87.62) | 288 (89.16) | 0.05 |
| Dynamic compliance (ml/cmH2O), mean ± SD | 33.3 ± 24.1 | 32.9 ± 26.8 | 0.01 |
| PaO_2_/FiO_2_ ratio (mmHg), mean ± SD | 180.33 ± 56.62 | 180.62 ± 49.84 | 0.02 |
| Total respiratory rate (breaths/min), mean ± SD | 21.5 ± 6.3 | 21.4 ± 7.0 | 0.01 |
|  |  |  | p-value |
| **Variables not included in propensity score estimation** |  |  |  |
| FiO_2_, median (q_1_; q_3_) | 0.45 (0.40 ; 0.60) | 0.80 (0.65 ; 1.00) | <.0001 |
| PaO_2_ (mmHg), mean ± sd | 81.6 ± 11.2 | 145.6 ± 42.5 | <.0001 |
| **Clinical Outcomes** |  |  |  |
| Hospital mortality (90 days) | 126 (39.01) | 136 (42.11) | 0.4713 |
| Ventilation free days (days), median (q_1_; q_3_) | 15.0 (0.0 ; 24.0) | 12.0 (0.0 ; 23.0) | 0.1674 |
| Duration mechanical ventilation (days), median (q_1_; q_3_) | 7.0 (3.0 ; 14.0) | 7.0 (4.0 ; 13.0) | 0.5458 |

*Abbreviations: ARDS: acute respiratory distress syndrome; BMI: body mass index; COPD: chronic obstructive pulmonary disease; FiO_2_: fraction of inspired oxygen; P_a_O_2_: arterial oxygen partial pressure; q_1_: first quartile; q_3_: third quartile; SD: standard deviation; SOFA: sepsis-related organ failure assessment.*

*Notes: 1) Normoxemia defined as 55 mmHg ≤ PaO_2_ ≤ 100 mmHg on day 1 of ARDS. 2) Excess oxygen use defined as P_a_O_2_ > 100 mmHg and FiO_2_ ≥ 0.60 on day 1 of ARDS.*
